# Electrical brain activity during human walking with parametric variations in terrain unevenness and walking speed

**DOI:** 10.1162/imag_a_00097

**Published:** 2024-02-22

**Authors:** Chang Liu, Ryan J. Downey, Jacob S. Salminen, Sofia Arvelo Rojas, Natalie Richer, Erika M. Pliner, Jungyun Hwang, Yenisel Cruz-Almeida, Todd M. Manini, Chris J. Hass, Rachael D. Seidler, David J. Clark, Daniel P. Ferris

**Affiliations:** a J. Crayton Pruitt Family Department of Biomedical Engineering, University of Florida, Gainesville, FL, United States; b McKnight Brain Institute, University of Florida, Gainesville, FL, United States; c Department of Neurology, University of Florida, Gainesville, FL, United States; d Department of Community Dentistry and Behavioral Science, University of Florida, Gainesville, FL, United States; e Pain Research and Intervention Center of Excellence (PRICE), University of Florida, Gainesville, FL, United States; f Department of Epidemiology, University of Florida, Gainesville, FL, United States; g Department of Health Outcomes and Biomedical Informatics, University of Florida, Gainesville, FL, United States; h Department of Applied Physiology and Kinesiology, University of Florida, Gainesville, FL, United States; i Norman Fixel Institute for Neurological Diseases, University of Florida, Gainesville, FL, United States; j Brain Rehabilitation Research Center, Malcom Randall VA Medical Center, Gainesville, FL, United States

**Keywords:** Electrocortical, balance, uneven terrain, EEG, walking speed

## Abstract

Mobile brain imaging with high-density electroencephalography (EEG) can provide insight into the cortical processes involved in complex human walking tasks. While uneven terrain is common in the natural environment and poses challenges to human balance control, there is limited understanding of the supraspinal processes involved with traversing uneven terrain. The primary objective of this study was to quantify electrocortical activity related to parametric variations in terrain unevenness for neurotypical young adults. We used high-density EEG to measure brain activity when 32 young adults walked on a novel custom-made uneven terrain treadmill surface with four levels of difficulty at a walking speed tailored to each participant. We identified multiple brain regions associated with uneven terrain walking. Alpha (8 – 13 Hz) and beta (13 – 30 Hz) spectral power decreased in the sensorimotor and posterior parietal areas with increasing terrain unevenness while theta (4 – 8 Hz) power increased in the mid/posterior cingulate area with terrain unevenness. We also found that within stride spectral power fluctuations increased with terrain unevenness. Our secondary goal was to investigate the effect of parametric changes in walking speed (0.25 m/s, 0.5 m/s, 0.75 m/s, 1.0 m/s) to differentiate the effects of walking speed from uneven terrain. Our results revealed that electrocortical activities only changed substantially with speed within the sensorimotor area but not in other brain areas. Together, these results indicate there are distinct cortical processes contributing to the control of walking over uneven terrain versus modulation of walking speed on smooth, flat terrain. Our findings increase our understanding of cortical involvement in an ecologically valid walking task and could serve as a benchmark for identifying deficits in cortical dynamics that occur in people with mobility deficits.

## 1. INTRODUCTION

Human bipedal locomotion is inherently unstable. Even when walking on a smooth, level surface, the body’s center of mass moves outside of the base of support and thus poses a challenge to maintain dynamic balance. The natural environment surrounding us is rarely flat. Walking over uneven terrain poses additional challenges to bipedal locomotion as people need to be aware of the external environment (e.g., irregular rocks, grass, slippery surfaces) and actively control their balance to prevent falls. Prior studies have found changes in biomechanical measures when walking over uneven terrain compared to flat terrain, including an increase in kinematic variability, a decrease in gait stability, and an increase in energetic cost (Coleman et al., 2016; Downey et al., 2022; Kent et al., 2019; Thomas et al., 2020; Voloshina & Ferris, 2015).

While walking on a flat surface is usually considered automatic and requires little contribution from the brain (Clark, 2015; Lau et al., 2014), performing complex walking tasks requires top-down cortical control at higher-order brain centers (reviewed by Fettrow et al. (2021)). Cognitive processes such as motor planning, motor execution, and error detection are necessary for walking over uneven terrain and avoiding obstacles (Clark et al., 2014; Clark et al., 2014; Hawkins et al., 2017). For example, when walking over some types of uneven terrain, people often plan foot placement based on visual information (Darici & Kuo, 2022; Matthis et al., 2018). People actively monitor their motor performance using multi-sensory feedback to generate corrective responses to control their balance. Traditionally, there has been a lack of brain imaging techniques enabling high-quality brain data during human locomotion.

The recent development of mobile imaging techniques using high-density electroencephalography (EEG) has enabled direct, non-invasive measurement of cortical activity during whole-body movement with millisecond temporal resolution. For example, there is building evidence for consistent changes in electrocortical activity with challenges to balance control (Bruijn et al., 2015; Jacobsen et al., 2022; Luu et al., 2017; Sipp et al., 2013; Solis-Escalante et al., 2021; Varghese et al., 2014). Compared to normal walking on a smooth, level surface, alpha (8 – 13 Hz) and beta (13 – 30 Hz) spectral power at the sensorimotor area was lower when walking on a ramp (Luu et al., 2017), walking on a narrow beam (Sipp et al., 2013), and walking on uneven grass terrain (Jacobsen et al., 2022). Beta spectral power at the premotor area was lower when participants were not given external stability support during walking (Bruijn et al., 2015). Additionally, theta (4 – 8 Hz) spectral power was greater during narrow beam walking compared with treadmill walking (Sipp et al., 2013). Theta power increased sharply when participants stepped off the balance beam (Peterson & Ferris, 2018; Sipp et al., 2013; Varghese et al., 2014). Each of these EEG spectral power bands (theta, alpha, beta) provide unique insight into brain function during motor tasks (reviewed by Cevallos et al. (2015)). Taken together, these results suggest that an increase in theta power and a decrease in alpha and beta power are associated with increasing demand for balance control during gait. However, few studies have investigated how electrocortical activities change during walking with parametrically varied complexity, which could lead to a better understanding of neural compensation in populations with mobility deficits for future studies (Fettrow et al., 2021).

In many studies examining balance control of walking, an important confounding factor across studies is walking speed (Downey et al., 2022; Liu et al., 2022). Faster walking speeds require greater muscle activation, mechanical energy, and metabolic energy expenditure (Franz & Kram, 2013; Neptune et al., 2008). There is mixed evidence as to whether increasing walking speed increases, decreases, or does not alter gait stability (Hak et al., 2013; Kang & Dingwell, 2008; Li et al., 2005). Two previous studies found that alpha and beta band spectral power at sensorimotor and posterior parietal areas was lower for faster walking speeds compared to slower walking speeds (Bulea et al., 2015; Nordin et al., 2020). Yet, both studies had relatively small sample sizes and did not include slow walking speeds (i.e., <0.5 m/s).

The primary goal of this study was to determine how electrocortical activity measured by EEG changed with parametric changes in terrain unevenness for neurotypical young adults. We used a high-density EEG system to measure brain activities when young adults walked on a novel uneven terrain treadmill surface with four levels of difficulty at a walking speed tailored to each participant. We hypothesized that alpha and beta spectral power would be lower with greater terrain unevenness at the sensorimotor and posterior parietal area due to the increasing demand for precise foot placement and balance control over uneven terrain. We also hypothesized that theta spectral power would be greater with greater terrain unevenness in the anterior cingulate area due to the increasing demand to monitor motor performance. Related to our hypotheses, we expected to find greater intra-stride spectral power fluctuations on more uneven terrains due to more cortical processing of sensorimotor information and motor adjustments within the gait cycle. The secondary goal was to determine how electrocortical activity measured by EEG changed with walking speed. We hypothesized that alpha and beta spectral power would be lower at the sensorimotor and posterior parietal areas at slower speeds. Intra-stride spectral power fluctuations would be greater in the alpha and beta power band at slower walking speeds because of the need to consciously adapt to the required slow walking speed. In addition to testing our main hypotheses, we performed an exploratory analysis on other brain areas such as supplementary motor area, occipital area for uneven terrain, and speed effects. The results from this study will increase our understanding of brain activity during an ecologically valid uneven terrain walking task and serve as a benchmark for future studies on population with mobility deficits.

## 2. MATERIALS AND METHODS

### 2.1. Participant

We recruited a total of 35 healthy young individuals (19 females, mean age 24 +/− 4 yrs, walking speed on uneven terrain = 0.7 +/− 0.2 m/s) with no musculoskeletal, severe cardiovascular, orthopedic, or psychiatric diagnosis as part of a larger parent study (i.e., the Mind in Motion study (NCT03737760)). Full inclusion and exclusion criteria were reported by Clark et al. (2020). Three participants reported being left-handed. A recent paper using EEG for mobile brain imaging found a Cohen’s d of 1.22 for comparing electrocortical power fluctuations between walking on a paved concrete surface and a grassy unpaved surface (Jacobsen et al., 2022). Based on that effect size and their data, we aimed for 30 participants so that we would have a minimum of 15 participants’ data in each independent component cluster for EEG analysis. We recruited 35 participants considering a ~10% – 15% drop-out rate due to gelling or noise artifacts based on previous studies in our lab (Jacobsen & Ferris, 2023; Studnicki & Ferris, 2023). We removed one female participant from the analysis due to difficulty with electrode gelling during data collection. We also removed two female participants due to technical issues with the data. All participants provided informed consent before participating in the experiment. The study was conducted in accordance with the Declaration of Helsinki and approved by the Institutional Review Board of the University of Florida (IRB 201802227).

### 2.2. Design of uneven terrain treadmill

**Fig. 1.**
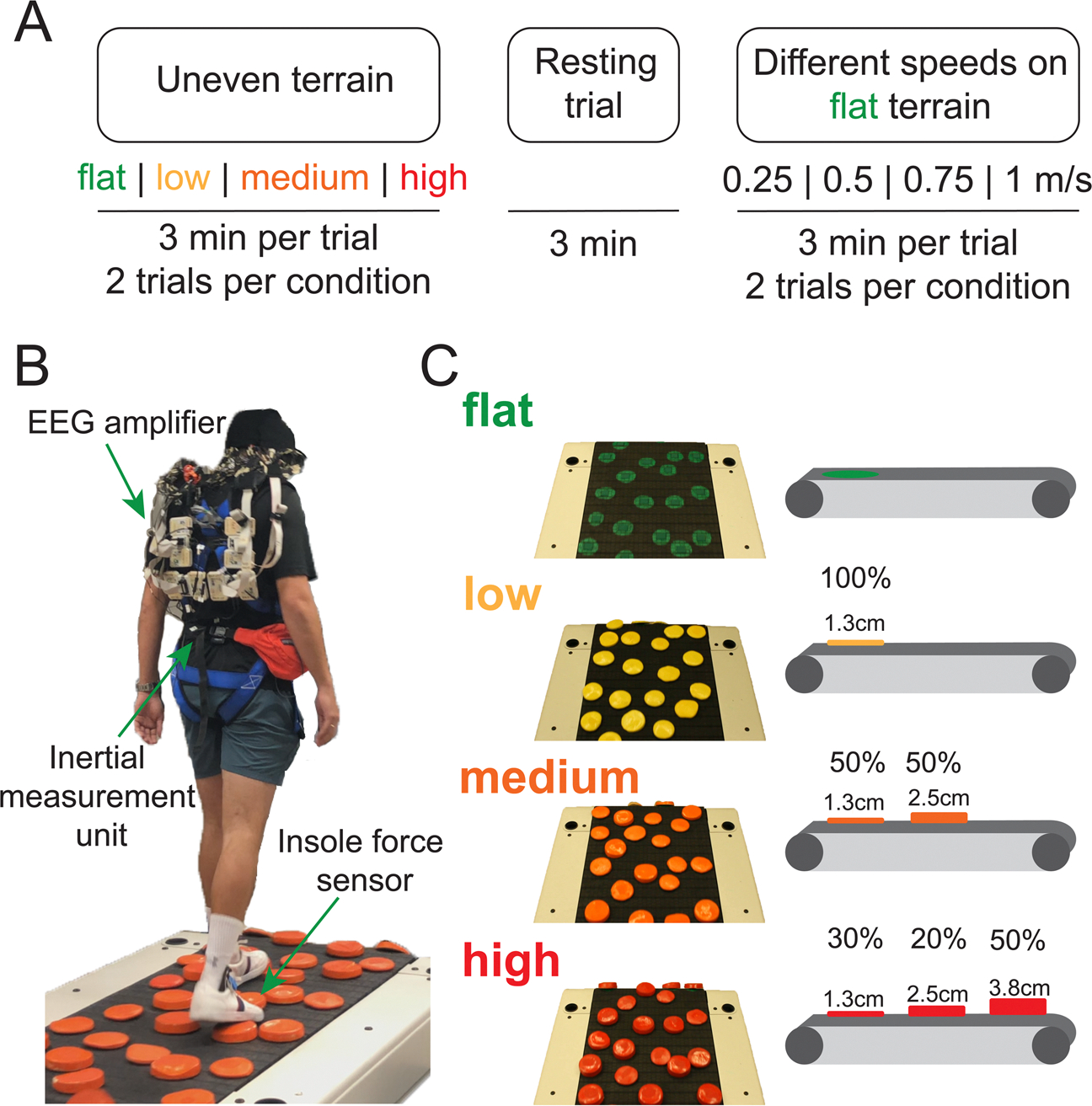
Experimental protocol and setup. (A) Participants completed treadmill walking trials on four different levels of uneven terrain (flat, low, medium, high), one seated resting trial, and walking trials at four different speeds (0.25 m/s, 0.50 m/s, 0.75 m/s, 1.0 m/s) performed on the flat terrain. Participants completed a block of two treadmill walking trials per condition. Each trial was 3 minutes. (B) Experimental setup. (C) Design of uneven terrain treadmill. Low, medium, and high terrain unevenness was achieved by altering the height of the rigid disks on the treadmill. Percentage refers to the proportion of disks that were the specified height.

Details of the terrain design have been described previously (Downey et al., 2022). We modified the terrain unevenness using rigid foam disks that were attached to the slat belt treadmill (PPS 70 Bari-Mill, Woodway, Waukesha, WI, USA; 70 × 173 cm walking surface; Fig. 1). The rigid disks were made from polyurethane using a circular free-rise mold with a diameter of 12.7 cm. Hoop-and-loop fasteners attached the disks to the treadmill surface so that we could easily switch the level of terrain unevenness. The spatial configuration of the disks was the same for each uneven terrain and consistent across participants. There were no large gaps between disks, so participants stepped on at least one disk with almost all footfalls.

We parametrically varied the terrain unevenness for low, medium, and high levels by altering the height of the rigid disks on the treadmill. The low terrain included yellow 1.3 cm high disks. The medium terrain included orange disks of two different heights: 50% of them were 1.3 cm high disks, and 50% were 2.5 cm high. The high terrain consisted of red disks of three different heights: 30% were 1.3 cm tall, 20% were 2.5 cm tall, and 50% were 3.8 cm tall. There were no disks for the flat terrain, but there were painted green circles on the treadmill in the same configuration as the other terrains.

### 2.3. Experimental protocol

The experimental protocol is a subset of the Mind in Motion larger study. Details of the full protocol were provided in Clark et al. (2020). We included one session of EEG and one session of MRI scans for this experiment protocol. For 21 out of 35 participants, the EEG and MRI sessions were performed on separate days within ~30 days or less of each other. Ten of our MRI scans were collected about two months later than EEG. Four of our MRI scans were collected about six months to a year later than the EEG visit due to shutdown of our research facility during COVID-19. Since there is usually no substantial brain structural change occurring within six months in younger adults (Hedman et al., 2012), any minor brain structural changes would be very unlikely to impact our source localization analysis with younger adults.

The EEG visit included treadmill walking trials on four different levels of uneven terrain (flat, low, medium, and high), treadmill walking at four different speeds (0.25 m/s, 0.5 m/s, 0.75 m/s, 1.0 m/s) on the flat surface, and one seated resting trial (Fig. 1A). Before the EEG visit, participants walked on an overgr*o*und version of the flat, low, medium, and high terrain on a 3.5 meter mat three times. We instructed the participants to walk at a normal, comfortable pace. The overground speed for each terrain was computed as the average speed to walk through the middle 3-meter portion. We set the treadmill walking speed across all terrains to 75% of the slowest overground speed (slowest terrain) because the treadmill walking speed was ~10% – 15% slower than the overground walking speed and due to safety concerns for uneven terrain walking (Dal et al., 2010; Malatesta et al., 2017). Participants wore a harness to prevent falling to the ground, but the harness did not provide any body weight support unless they fell. We did not record any falls in this dataset. Participants performed a block of two treadmill walking trials per condition. Each walking trial was 3 minutes. We pseudorandomized the conditions with 8 unique orders of uneven terrain conditions and speed conditions, respectively. The total amount of data collected including both walking trials and the resting trial was ~50 minutes.

### 2.4. Data acquisition

**Fig. 2.**
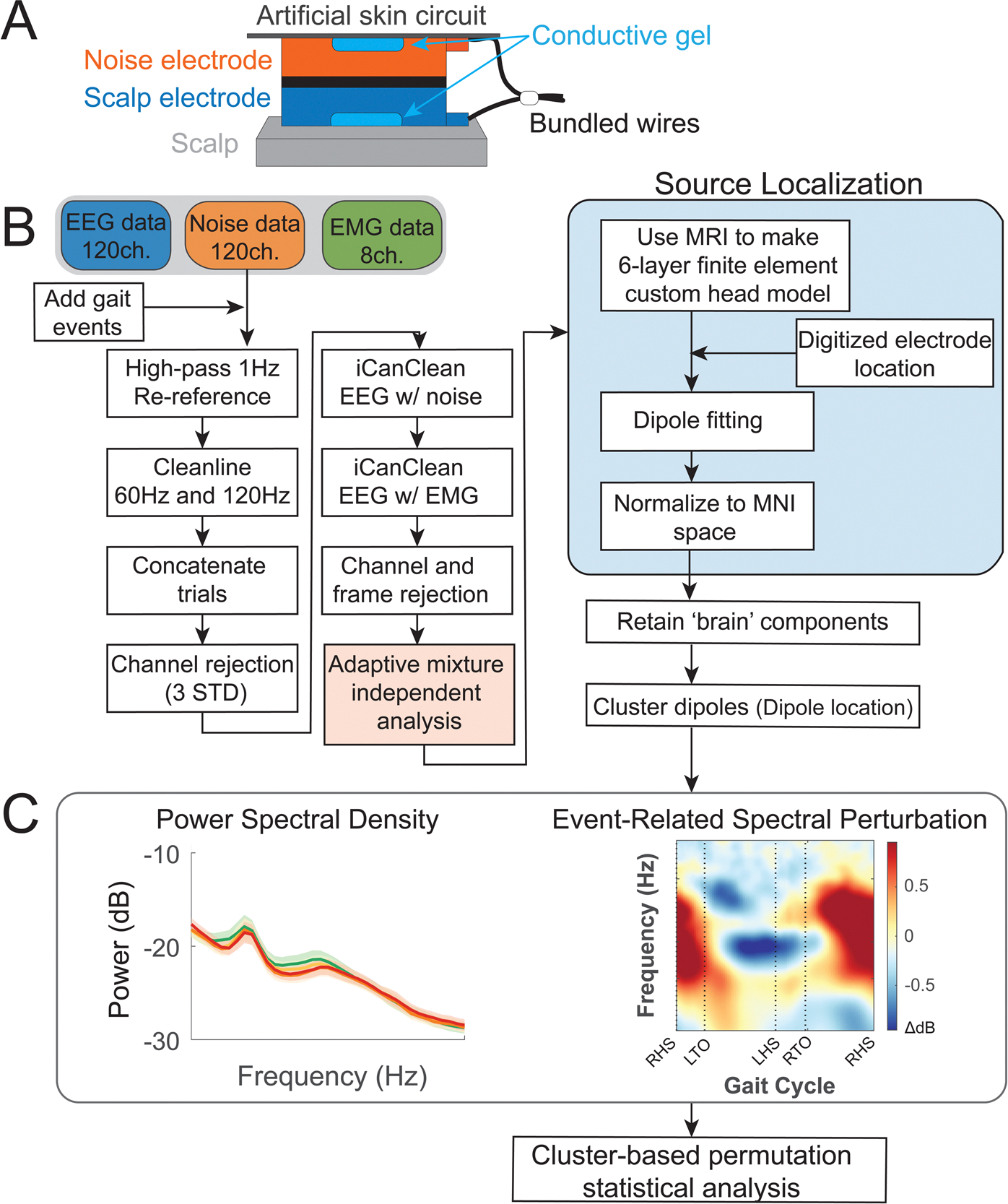
EEG processing pipeline. (A) Dual-electrode EEG setup. Scalp electrodes and noise electrodes were mechanically coupled. We used a conductive fabric as an artificial skin circuit to bridge the noise electrodes. Figure is by courtesy of Amanda Studnicki. (B) Data processing flowchart with steps for EEG pre-processing, source localization, and clustering brain components. (C) We performed the analysis in the frequency domain after clustering brain components to investigate how electrocortical activity changes with terrain unevenness and speed. We averaged power spectral density (PSD) and event-related spectral perturbations (ERSPs) tied to gait events (RHS: right heel strike; LTO: left toe off; LHS: left heel strike; RTO: right toe off) within each brain cluster.

During the EEG visit, participants wore a custom-made dual-layer EEG cap (ActiCAP snap sensors; Brain Products GmbH, Germany), including 120 scalp electrodes, 120 mechanically coupled noise electrodes, and 8 electrodes to measure muscle activity. The scalp electrodes followed a 10–05 electrode system. We re-purposed eight of the original 128 scalp electrodes (TP9, P9, PO9, O9, O10, PO10, P10, and TP10) to measure muscle activity of the sternocleidomastoid and trapezius on the left and right sides and thus 120 electrodes were placed on participants’ scalp. We inverted and mechanically coupled noise electrodes to the scalp electrodes (Fig. 2A) (Nordin et al., 2018; Studnicki et al., 2022). We used a conductive fabric as an artificial skin circuit and bridged the noise electrodes. We aimed to keep all scalp electrode impedance values below 15 Kohm during the setup. Ground and reference electrodes were kept below 5 Kohm. We digitized the electrode locations using a structural scanner (ST01, Occipital Inc., San Francisco, CA). We used four LiveAmp 64 amplifiers and logged EEG data at 500 Hz. The online reference and ground electrodes were at CPz and Fpz, respectively.

In addition, we recorded the ground reaction force of each foot with capacitive shoe insole sensors (loadsol 1–184 sensor, Novel Electronics Inc., St. Paul, MN, USA) at 200 Hz and sacrum kinematics with an inertial measurement unit (IMU, Opal APDM Inc., Portland, OR, USA) at 128 Hz. We synchronized data from the IMU to the EEG LiveAmp offline via a pulse at the beginning and the end of the trial. We also synchronized the insole sensor force data with EEG LiveAmp offline via pulses occurring every five seconds. More details about the data synchronization were provided in our previous paper (Downey et al., 2022).

### 2.5. MRI acquisition

On a separate day, we collected structural MRI scans for all participants. We acquired the anatomical brain structure from a T1-weighted sequence. The parameters for this anatomical image were: repetition time (TR) = 2000 ms, echo time (TE) = 2.99 ms, flip angle = 8°, voxel resolution = 0.8 mm^3^, and field of view = 256 × 256 × 167 mm^2^ (4:22 minutes of scan time), using a 64-channel coil array on a 3 T Siemens MAGNETOM Prisma MR scanner.

### 2.6. Data processing

#### 2.6.1. Behavioral analysis

We post-processed the kinematic and kinetic data in Matlab 2020b (Mathworks, USA) to compute the variables of interest. We defined foot strike as the point when ground reaction forces became greater than 20 N and foot off as the point when ground reaction forces became less than 20 N (Downey et al., 2022). We defined the step duration as the time between consecutive foot strikes. We also computed the peak-to-peak excursion of the sacrum in the anteroposterior and mediolateral direction using the IMU data. Details of the algorithm and calculations were reported in our recent paper (Downey et al., 2022). We removed outliers that were +/−2.5 standard deviations away from the mean (Downey et al., 2022). We calculated the variability of each of these measures as the coefficient of variation (standard deviation over mean).

#### 2.6.2. EEG data pre-processing

We processed all EEG data using custom Matlab scripts (R2020b) and EEGLAB (v 2021.0; Fig. 2B) (Delorme & Makeig, 2004). We first applied a 1 Hz high-pass filter (−6 dB at 0.5 Hz) with *eegfiltnew* on all scalp, noise, and muscle channels to remove drift for each trial. We also applied a 20 Hz high-pass filter with *eegfiltnew* on muscle channels. We used the CleanLine plugin in EEGLAB to remove line noise at 60 Hz and 120 Hz. We rejected bad channels that were 3 standard deviations away from the mean of EEG and noise channels, respectively. We performed average reference for scalp, noise, and muscle channels respectively.

We then used a novel algorithm iCanClean (Downey & Ferris, 2023; Gonsisko et al., 2023) to remove artifacts that were highly correlated with noise reference electrodes (R^2^ = 0.65 with a four-second moving window) and muscle reference electrodes (R^2^ = 0.4 with a four-second moving window). This algorithm has been previously validated to improve mobile EEG data quality during human experiments (Gonsisko et al., 2023; Studnicki et al., 2022). Then, we used *clean_artifacts* in EEGLAB to remove bad channels and noisy time frames using default parameters except for the following parameters (chan_crit1 = 0.5, win_crit1 = 0.4, win-Tol = [−Inf, 10]). These parameters were selected in a preliminary analysis of a subset of the data, which aimed to minimize the number of channels and time frames rejected while maximizing a good number of brain components by ICLabel (Liu et al., 2023; Pion-Tonachini et al., 2019). We retained 110 ± 6 channels and rejected a maximum of 5% of time frames (mean = 1%). Scalp EEG data were re-referenced again. We performed adaptive mixture independent component analysis (AMICA) on the preprocessed data (Palmer et al., 2011) to decompose the preprocessed EEG data into statistically independent components. We later used the independent components to perform source localization.

#### 2.6.3. Individual-specific volume conduction model

We processed the T1-weighted MRI using Fieldtrip (v.20210910) for each participant (Liu et al., 2023). The images were resliced to be isotropic (1 mm^3^). We digitized the fiducial locations (left/right preauricular, nasion) on the MRI. We used the *headreco* from SimNIBS toolbox (v 3.2) to perform tissue segmentation (Nielsen et al., 2018). We segmented individual MRIs into six tissue layers (scalp, skull, air, cerebrospinal fluid, gray matter, and white matter). Hexahedral meshes were generated with recommended node-shift parameters using *prepare_mesh_hexahedral.* We co-registered the digitized electrode locations to the individual-specific head model by aligning the fiducial locations digitized in the MRIs to those in the structural scan. We calculated the leadfield matrix for each individual-specific head model using the SIMBIO toolbox in Fieldtrip. We distributed source positions in the gray matter 5 mm apart.

#### 2.6.4. Source localization

We performed EEG source localization using an equivalent dipole fitting approach using *ft_dipolefitting* function in the Fieldtrip toolbox with the individual-specific volume conduction head models. We then converted the dipole locations to the Montreal Neurological Institute (MNI) template. We retained brain components using the following criteria (Liu et al., 2023): 1) ICLabel (Pion-Tonachini et al., 2019) (version: lite) classified the brain probability of greater or equal to 50%, 2) negative slope of the power density spectrum for 2 – 40 Hz to remove muscle components, 3) residual variance of dipole fitting <15%, 4) minimal high-frequency power coupling using PowPowCAT toolbox to further remove muscle components (Thammasan & Miyakoshi, 2020), and lastly, we visually inspected all the components and removed non-brain components based on previous criteria. We retained 14 ± 5 brain components per participant.

#### 2.6.5. K-means clustering of brain components

We clustered the brain components by dipole location using k-means in EEGLAB. We determined the optimal number of clusters (k = 12) using Silhoutte and Davies-Boukin criteria in *evalcluster*. We retained the clusters with more than half of the participants. Components that are further than three standard deviations away from any of the clusters were identified as outliers. In the case that multiple components per subject existed in a cluster, we selected the component with the lowest residual variance to prevent artificially inflating the sample size (Studnicki & Ferris, 2023).

#### 2.6.6. Computing power spectral density and event-related spectral perturbations

We then performed frequency and time-frequency analyses for each cluster. For the walking trials, data were segmented into epochs of 5 seconds (from 0.5 seconds before to 4.5 seconds after the right foot strike). The epoch length was chosen to accommodate participants with long step durations during the slowest walking condition (0.25 m/s). We rejected epochs that were three standard deviations from the mean gait event time and 10% of epochs that had the highest voltage maximal, resulting in 205 ± 30 epochs for flat, 202 ± 31 epochs for low, 205 ± 30 epochs for medium, 208 ± 37 epochs for high terrain, and 108 ± 18 epochs for 0.25 m/s, 170 ± 17 epochs for 0.5 m/s, 210 ± 23 epochs for 0.75 m/s, and 249 ± 18 for 1.0 m/s. We found fewer epochs at slower speeds as the number of steps reduced for a trial with fixed duration.

For the frequency analysis, we computed the log power spectral density (PSD) using *spectopo* from EEGLAB with default parameters for independent components in each cluster and normalized by subtracting each individual’s mean log spectral power density from all conditions. We used the FOOOF toolbox (Donoghue et al., 2020) to separate the aperiodic and periodic components of each power spectra from 3 Hz to 40 Hz (peak width limits: [1 8], minimum peak height: 0.05, maximum number of peaks: 2). We computed the flattened power spectral density by subtracting the aperiodic component from each of the original power spectral density. Lastly, we computed average power for each band using the flattened power spectral density.

We then assessed the electrocortical activity tied to gait events using event-related spectral perturbations (ERSPs). We computed the single trial spectrograms with *newtimef* (Morlet wavelets cycles: [3 0.8]). ERSPs were time-warped to the gait cycle from right foot-strike to the subsequent right foot-strike. ERSPs were normalized to 1) average spectral power across all gait cycles within conditions and 2) average spectral power across gait cycles for all conditions (common baseline removal). We averaged ERSPs for each participant and then averaged across all participants for each cluster and for each condition.

### 2.7. Statistical analysis

All statistical analyses were performed in Matlab 2020b (Mathworks). We first assessed whether there were significant differences in any behavioral measures across terrain using a linear mixed-effect model for each outcome measure. The dependent variables included step duration, step duration coefficient of variation, and sacral excursion coefficient of variation in mediolateral and anteroposterior directions. The independent variables included Terrain (flat, low, medium, high). Walking speed was included as a covariate for all mixed-effect models. We included a random intercept to account for unmodeled sources of between-subject variability. We calculated the conditional R^2^ which is the proportion of variance explained by both the fixed and random effects for each linear mixed effect model. We also calculated Cohen’s f^2^ as a measure of effect size for the main effects and used the following definitions for the effect sizes: 0.02 = small, 0.15 = medium, and 0.35 = large effect size (Cohen, 2013). We analyzed the residual normality using the Lilifores Test. Pairwise comparison was corrected for multiple comparison using false discovery rate (Benjamini & Hochberg, 1995). Significance level was kept α < 0.05.

We then assessed if power spectral density for each cluster differed across terrain and speed, respectively. We performed non-parametric permutation statistics for both the original and flattened power spectral density using Fieldtrip in EEGLAB (α = 0.05, 2000 iterations) and corrected for multiple comparisons using false discovery rate. We also analyzed if average power for theta, alpha, and beta band for each cluster after removing the aperiodic component differed across terrain and speed using a linear mixed model for each dependent variable. The independent variables included Terrain or Speed. Terrain was modeled as a categorical variable while Speed was a continuous variable. We included a random intercept for each model. The reference level was set to flat condition or 1.0 m/s condition. We used 1.0 m/s as the reference level because this speed was closer to the self-selected walking speed in young adults (Liu et al., 2022; Nagano et al., 2013).

For ERSPs, we first assessed the statistically significant time-frequency change within each condition. The spectral baseline was the average spectral power across all gait cycles within the conditions. For each condition in each cluster, we bootstrapped ERSPs (α = 0.05, 2000 iterations) and corrected for multiple comparison using false discovery rate. We then assessed the differences in ERSPs across terrain and speed, respectively. We performed non-parametric permutation statistics to determine the time-frequency differences across terrain conditions and relative to flat condition (ERSP_terrain_ – ERSP_flat_). Similarly, we performed permutation statistics to determine time-frequency differences across speed conditions and relative to the 1.0 m/s condition (ERSP_speed_ – ERSP_1.0 m/s_). All these analyses were corrected with cluster-based multiple comparison using Fieldtrip through EEGLAB (α = 0.05, 2000 iterations).

## 3. RESULTS

### 3.1. Behavioral analysis

**Fig. 3.**
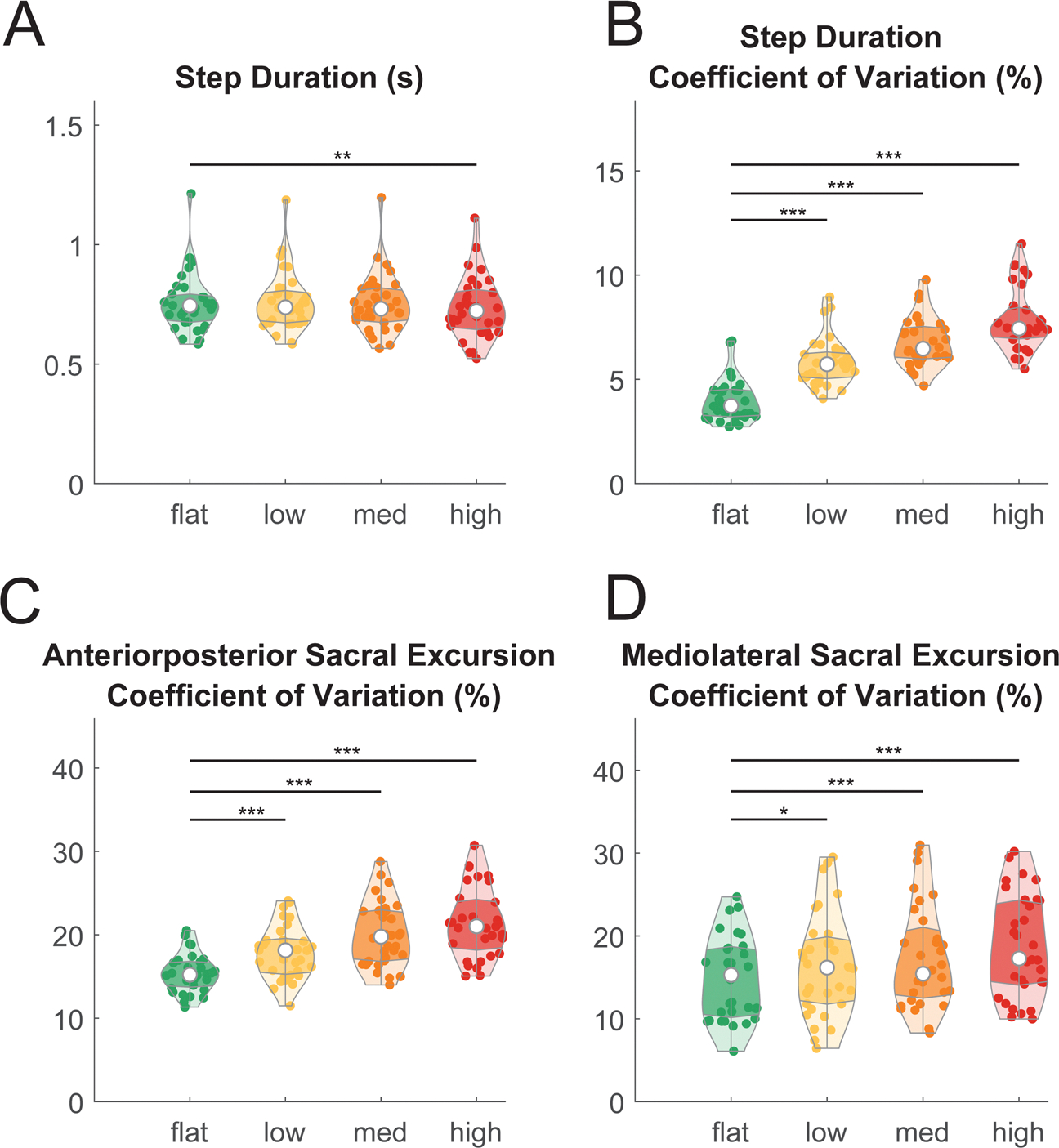
Violin plot shows the behavioral measures during walking on different levels of terrain unevenness and at different speeds. (A) Step duration time at flat, low, medium, and high terrain conditions. (B) Step duration coefficient of variation at different terrains. Sacral excursion coefficient of variation in the anteroposterior (C) and mediolateral (D) direction. The shaded regions represent data distribution across participants by estimating the probability density function. The white dots represent the median of data. The darker shaded region represents the 25 to 75 percentiles of the data. Individual data points are plotted as small dots. We only showed pairwise comparison statistics relative to the flat terrain. Refer to Table 1 for full pairwise comparison results (*pFDR_adjusted_ < 0.05, **pFDR_adjusted_ < 0.01, ***pFDR_adjusted_ < 0.001).

Behavioral results demonstrated that the novel, custom-made uneven terrain treadmill successfully increased gait kinematic variability, which may stem from reduced stability and changes in behavioral strategies to maintain balance when walking over uneven terrain, as compared to an even surface. We assessed the effect of terrain unevenness on behavioral measures, including step duration, step duration variability, and sacral excursion variability in the anteroposterior and mediolateral direction in young adults (n = 32; Fig. 3) using a linear mixed-effect model for each outcome measure. We found a significant main effect on step duration (F(3, 123) = 6.2, p < 0.001, Cohen’s f^2^ = 0.20; Fig. 3A) and step duration coefficient of variation (F(3, 123) = 213.6, p < 0.001, Cohen’s f^2^ = 8.7; Fig. 3B). Post-hoc pairwise comparison corrected by false discovery rate (FDR) indicated that longer step duration and greater step duration coefficient of variation were associated with greater terrain unevenness (Table 1).

We also found a significant main effect of terrain on sacral excursion coefficient of variation in both the anteroposterior direction (F(3, 123) = 37.2, p < 0.001, Cohen’s f^2^ = 1.51; Fig. 3C) and mediolateral direction (F(3, 123) = 14.1, p < 0.001, Cohen’s f^2^ = 0.51; Fig. 3D). Greater sacral excursion variability was associated with greater terrain unevenness in both anteroposterior and mediolateral directions (Table 1).

### 3.2. EEG source analysis

**Fig. 4.**
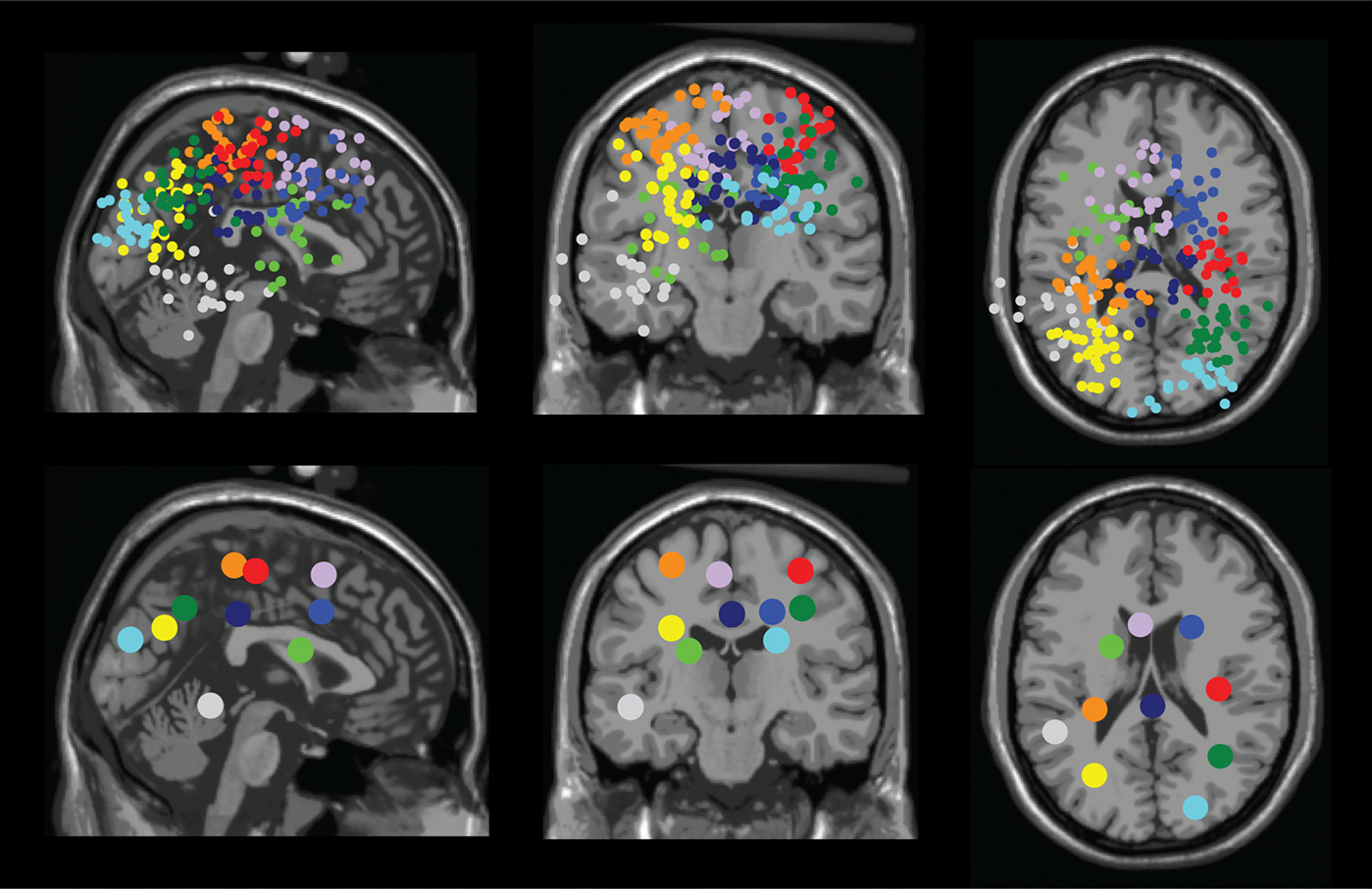
Dipole location for all participants (top row) and centroid of each cluster (bottom row) in sagittal (left), coronal (middle), and axial (right) planes. We identified clusters located at: right sensorimotor area (red), left sensorimotor area (orange), right premotor (medium blue), left pre-supplementary motor (purple), right posterior parietal (green), left posterior parietal (yellow), occipital area (cyan/light blue), mid/posterior cingulate (navy), caudate area (lime), and left temporal area (light gray).

We identified multiple neural sources that contained dipoles from more than half of the participants (n > 16). These dipole clusters were located at right sensorimotor (n = 21), left sensorimotor (n = 28), right premotor (n = 24), left pre-supplementary motor (n = 24), right posterior parietal (n = 28), left posterior parietal (n = 27), occipital (n = 20), mid/posterior cingulate (n = 24), caudate (n = 20), left temporal area (n = 17), and precuneus (Fig. 4, Table 2).

### 3.3. Terrain unevenness on EEG power spectral density

#### 3.3.1. Sensorimotor areas

**Fig. 5.**
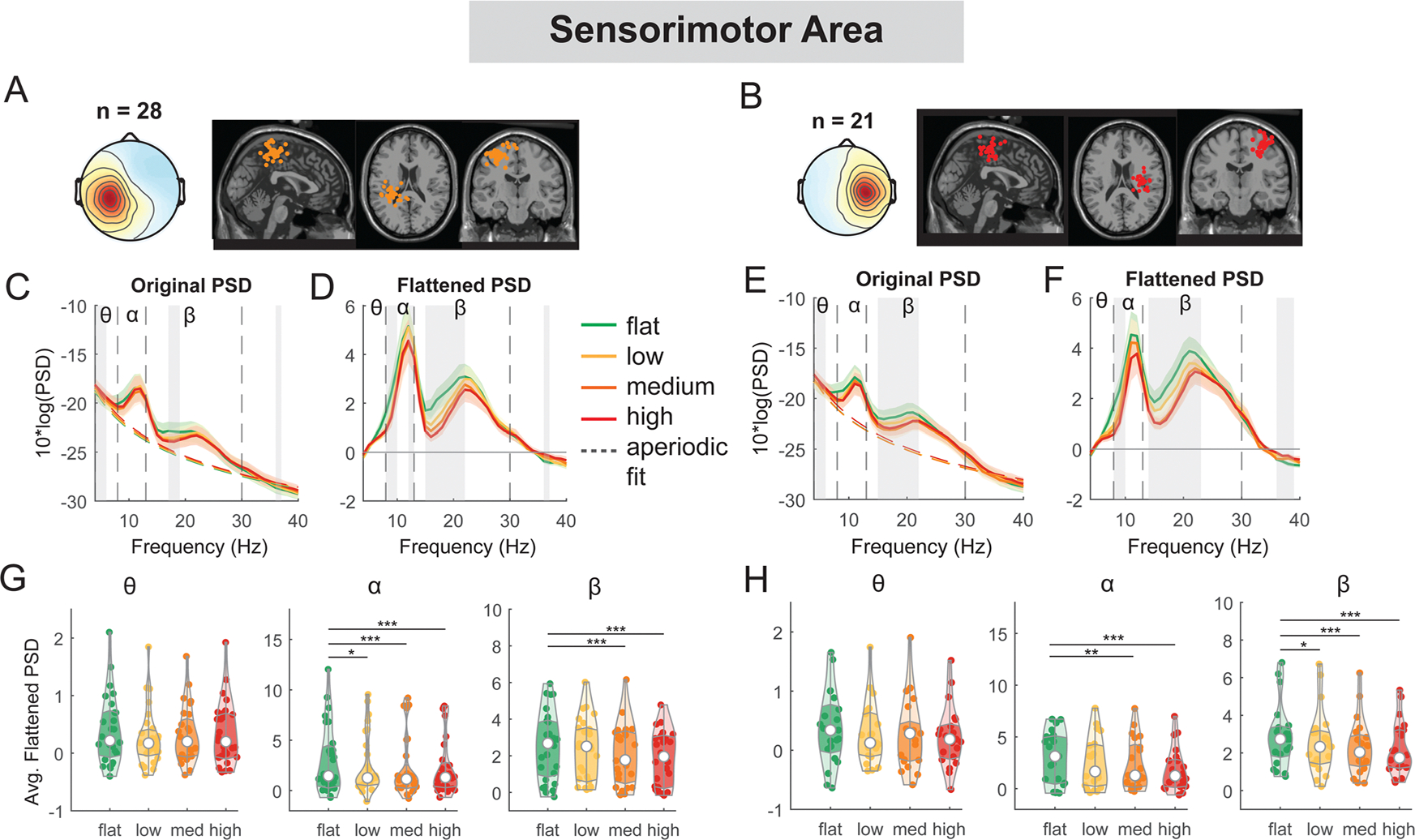
Power spectral density changes with terrain unevenness at the left and right sensorimotor area. (A, B) Average scalp topography within the cluster and dipole locations for each component plotted on the Montreal Neurological Institute template for the left and right sensorimotor cluster. Average original PSDs changed with terrain unevenness for the left (C) and right (E) sensorimotor cluster. Shaded colored areas indicated standard error of PSDs across components in the cluster. Dashed colored lines indicated average aperiodic fit. Gray shaded areas indicated a significant effect of terrain on PSDs. Vertical black dashed lines indicated main frequency bands of interest—theta (4 – 8 Hz), alpha (8 – 13 Hz), and beta (13 – 30 Hz). Average flattened PSDs after removing the aperiodic fit for the left (D) and right (F) sensorimotor cluster. (G - H) Average power for theta, alpha, and beta band computed from flattened PSDs for all components within the cluster for the left and right clusters. Black bars and asterisks (*) indicate a significant difference in average power compared to the flat conditions (*p < 0.05, **p < 0.01, ***p < 0.001).

**Fig. 6.**
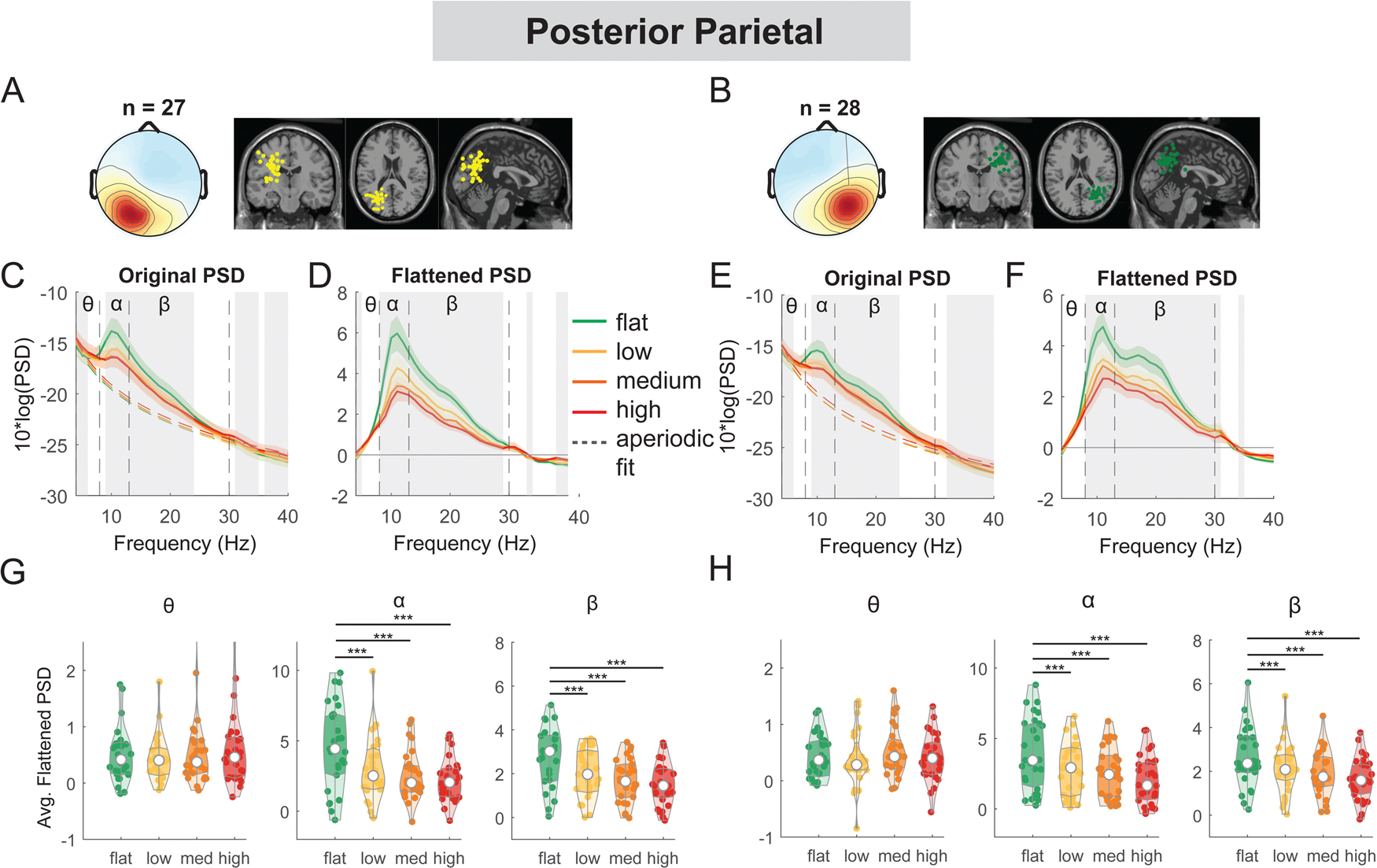
Power spectral density changes with terrain unevenness at the left and right posterior parietal area. (A, B) Average scalp topography within the cluster and dipole locations for each component plotted on the Montreal Neurological Institute template for the left and right posterior parietal cluster. Average original PSDs changed with terrain unevenness for the left (C) and right (E) posterior parietal clusters. Shaded colored areas indicated standard error of PSDs across components in the cluster. Dashed colored lines indicated average aperiodic fit. Gray shaded areas indicated a significant effect of terrain on PSDs. Vertical black dashed lines indicated main frequency bands of interest—theta (4 – 8 Hz), alpha (8 – 13 Hz), and beta (13 – 30Hz). Average flattened PSDs after removing the aperiodic fit for the left (D) and right (F) posterior parietal cluster. (G - H) Average power for theta, alpha, and beta band computed from flattened PSDs for all components within the cluster for the left and right clusters. Black bars and asterisks (*) indicate a significant difference in average power compared to the flat conditions (***p < 0.001).

**Fig. 7.**
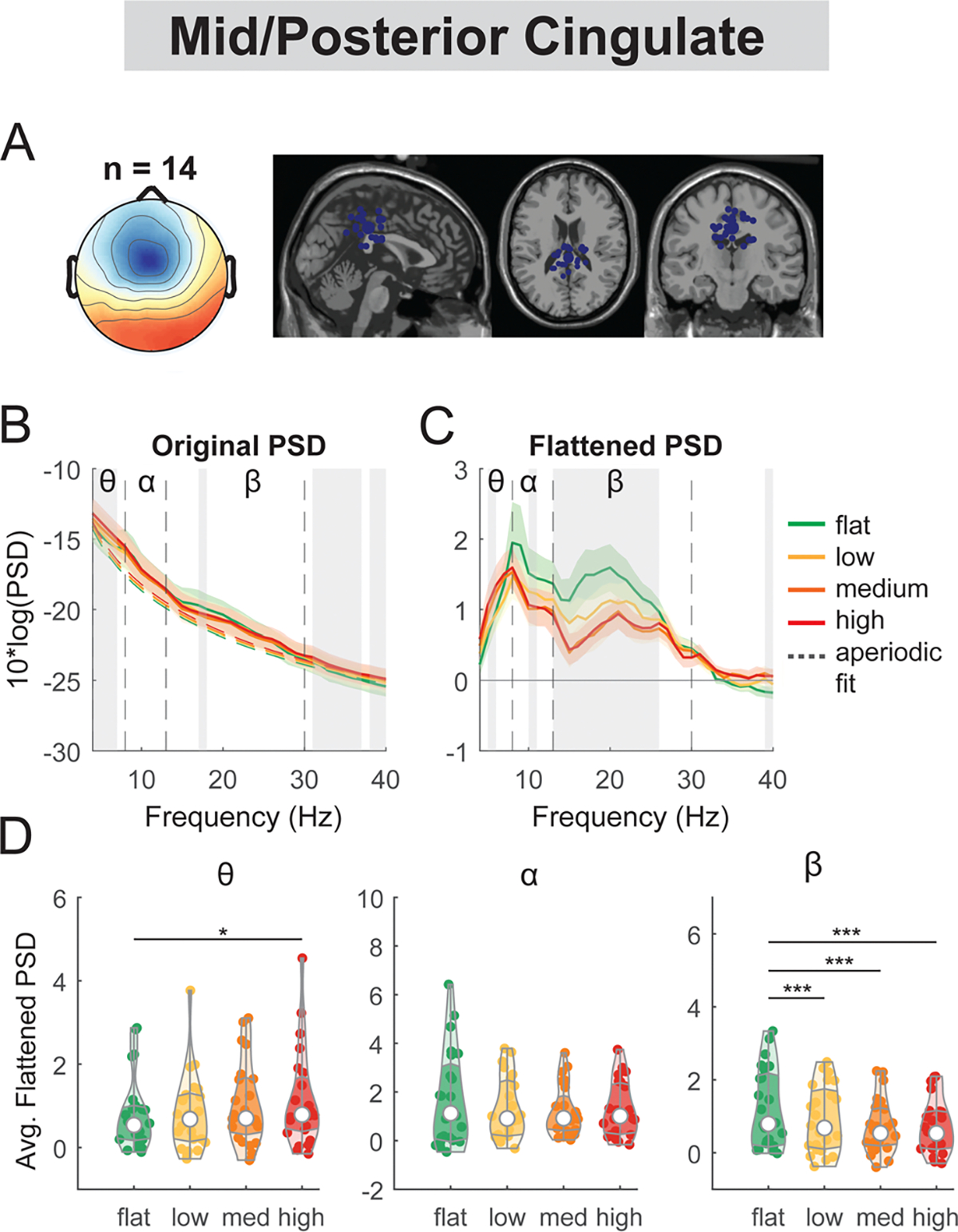
Power spectral density changes with terrain unevenness at the mid/posterior cingulate area. (A) Average scalp topography within the cluster and dipole locations for each component plotted on the Montreal Neurological Institute template for the mid/posterior cingulate cluster. (B) Average original PSDs changed with terrain unevenness for the cluster. Shaded colored areas indicated standard error of PSDs across components in the cluster. Dashed colored lines indicated average aperiodic fit. Gray shaded areas indicated a significant effect of terrain on PSDs. Vertical black dashed lines indicated main frequency bands of interest—theta (4 – 8 Hz), alpha (8 – 13 Hz), and beta (13 – 30 Hz). (C) Average flattened PSDs after removing the aperiodic fit. (D) Average power for theta, alpha, and beta band computed from flattened PSDs for all components within the cluster. Black bars and asterisks (*) indicate a significant difference in average power compared to the flat conditions (*p < 0.05, ***p < 0.001).

**Fig. 8.**
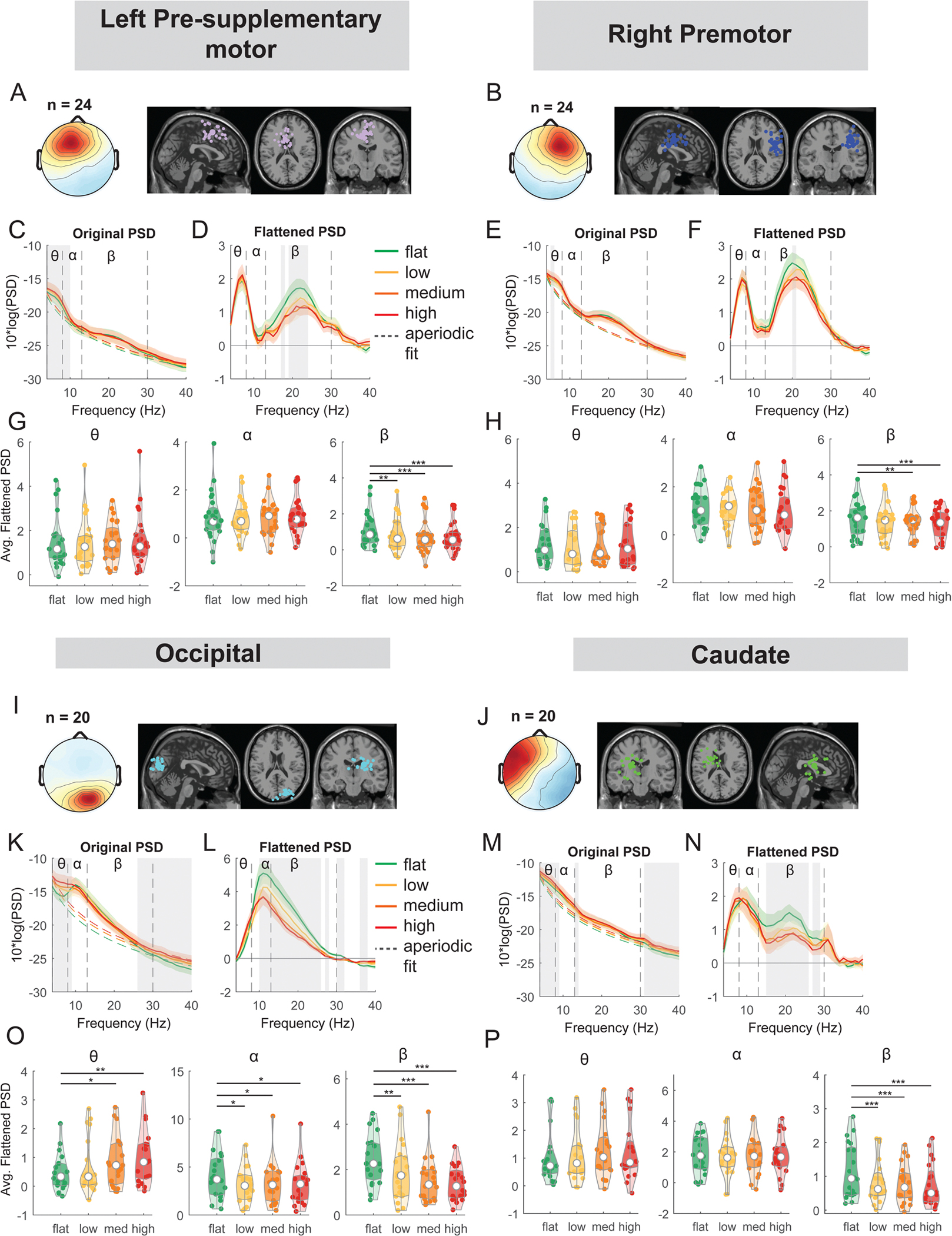
Power spectral density changes with terrain unevenness at the left pre-supplementary motor, right premotor area, occipital, and caudate area. (A) Average scalp topography within the cluster and dipole locations for each component plotted on the Montreal Neurological Institute template for the left pre-supplementary motor cluster. (C) Average original PSDs changed with terrain unevenness. Shaded colored areas indicated standard error of PSDs across components in the cluster. Dashed colored lines indicated average aperiodic fit. Gray shaded areas indicated a significant effect of terrain on PSDs. Vertical black dashed lines indicated main frequency bands of interest—theta (4 – 8 Hz), alpha (8 – 13 Hz), and beta (13 – 30 Hz). (D) Average flattened PSDs after removing the aperiodic fit. (G) Average power for theta, alpha, and beta band computed from flattened PSDs for all components within the left pre-supplementary motor cluster. Black bars and asterisks (*) indicate a significant difference in average power compared to the flat conditions (*p < 0.05, **p < 0.01, ***p < 0.001). (B) Average scalp topography within the cluster and dipole locations for the right premotor cluster. (E) Average original PSDs changed with terrain unevenness. (F) Average flattened PSDs after removing the aperiodic fit. (H) Average power for theta, alpha, and beta band computed from flattened PSDs for all components within the right premotor cluster. (I) Average scalp topography and dipole locations for the occipital cluster. (K) Average original PSDs changed with terrain unevenness. (L) Average flattened PSDs after removing the aperiodic fit. (O) Average power for theta, alpha, and beta band computed from flattened PSDs for all components within the occipital cluster. (J) Average scalp topography and dipole locations for the caudate cluster. (M) Average original PSDs changed with terrain unevenness. (N) Average flattened PSDs after removing the aperiodic fit. (P) Average power for theta, alpha, and beta band computed from flattened PSDs for all components within the caudate cluster.

We observed significant EEG power spectral modulation by terrain unevenness at multiple cortical areas (Figs. 5 – 8). At both left and right sensorimotor clusters, we found a main effect of terrain on average alpha power (left: F(3, 108) = 5.5, p = 0.001, Cohen’s f_2_ = 0.21; right: F(3, 80) = 6.7, p < 0.001, Cohen’s f_2_ = 0.35) and average beta power (left: F(3, 108) = 11.8, p < 0.001, Cohen’s f_2_ = 0.44; right: F(3, 80) = 12.5, p < 0.001, Cohen’s f_2_ = 0.63), with both alpha and beta power being lower on uneven terrain than on flat surface (Fig. 5). Compared to the flat condition, average alpha power was lower in the low (left: p = 0.02), medium (left: p < 0.001, right: p = 0.008), and high terrain conditions (left: p < 0.001, right: p < 0.001). Average beta power was also lower in the low (right: p = 0.012), medium (left: p < 0.001, right: p < 0.001), and high conditions (left: p < 0.001, right p < 0.001) compared to the flat condition.

#### 3.3.2. Posterior parietal areas

At both left and right posterior parietal clusters, there was a main effect of terrain on average alpha power (left: F(3, 104) = 19.3, p < 0.001, Cohen’s f^2^ = 0.90; right: F(3, 108) = 24.0, p < 0.001, Cohen’s f^2^ = 1.02) and average beta power (left: F(3, 104) = 23.8, p < 0.001, Cohen’s f^2^ = 1.11; right: F(3, 108) = 24.5, p < 0.001, Cohen’s f^2^ = 1.02). Lower alpha and beta power were both associated with greater terrain unevenness (Fig. 6). Compared to the flat condition, average alpha power was lower for low, medium, and high conditions (all p < 0.001 for left and right). Average beta power was also lower in low, medium, and high conditions (all p < 0.001 for left and right) as compared to the flat condition.

#### 3.3.3. Mid/posterior cingulate area

The mid/posterior cingulate cluster demonstrated a main effect of terrain on average theta power (F(3, 92) = 3.5, p = 0.019, Cohen’s f^2^ = 0.16) and beta band (F(3, 92) = 16.2, p < 0.001, Cohen’s f^2^ = 0.75; Fig. 7) but not on alpha power (F(3, 92) = 2.6, p = 0.055, Cohen’s f^2^ = 0.13). Theta power was greater for the high terrain condition (p = 0.01) compared to the flat terrain condition. Average beta power was lower in low (p < 0.001), medium (p < 0.001), and high conditions (p < 0.001) as compared to the flat condition.

#### 3.3.4. Premotor, pre-supplementary motor, occipital, and caudate areas

We also performed an exploratory analysis on the right premotor, left pre-supplementary motor, occipital, and caudate clusters. At the left pre-supplementary motor clusters, there was a main effect of terrain on average beta power (F(3, 92) = 8.2, p < 0.001, Cohen’s f^2^ = 0.37), with lower beta power associated with greater terrain unevenness (Fig. 8G). Compared to the flat condition, average beta power was lower in the low (p = 0.0070), medium (p < 0.001), and high conditions (p < 0.001). We also found an effect of terrain on average beta power (F(3, 92) = 5.3, p = 0.002, Cohen’s f^2^ = 0.24) at the right premotor cluster (Fig. 8H). Beta power was lower in the medium (p = 0.0061) and high (p < 0.001) terrain conditions.

A main effect of terrain was found on average theta power (F(3, 76) = 4.2, p = 0.0083, Cohen’s f^2^ = 0.25), alpha power (F(3, 76) = 3.1, p = 0.02, Cohen’s f^2^ = 0.19), and beta power (F(3, 76) = 12.8, p < 0.001, Cohen’s f^2^ = 0.82) at the occipital cluster (Fig. 8O). Theta power was greater in the medium (p = 0.01) and high terrain (p = 0.0015) conditions compared to the flat condition. Average alpha power was lower for low (p = 0.01), medium (p = 0.018), and high conditions (p = 0.016). Average beta power was lower for the low (p = 0.004), medium (p < 0.001), and high conditions (p < 0.001) as compared to the flat condition.

Lastly, we found a main effect of terrain on average beta power (F(3, 76) = 11.5, p < 0.001, Cohen’s f^2^ = 0.65) at the caudate cluster (Fig. 8P). Average beta power was lower for the low (p < 0.001), medium (p < 0.001), and high conditions (p < 0.001), compared to the flat condition.

### 3.4. Gait-related spectral perturbation during uneven terrain walking

We first computed the event-related spectral perturbations (ERSPs) tied to gait events with respect to average power at each frequency across the gait cycle of the same condition (Gwin et al., 2011). We unmasked the significant deviations from the average spectrum of each condition with a bootstrap method with false discovery rate multiple comparison correction.

**Fig. 9.**
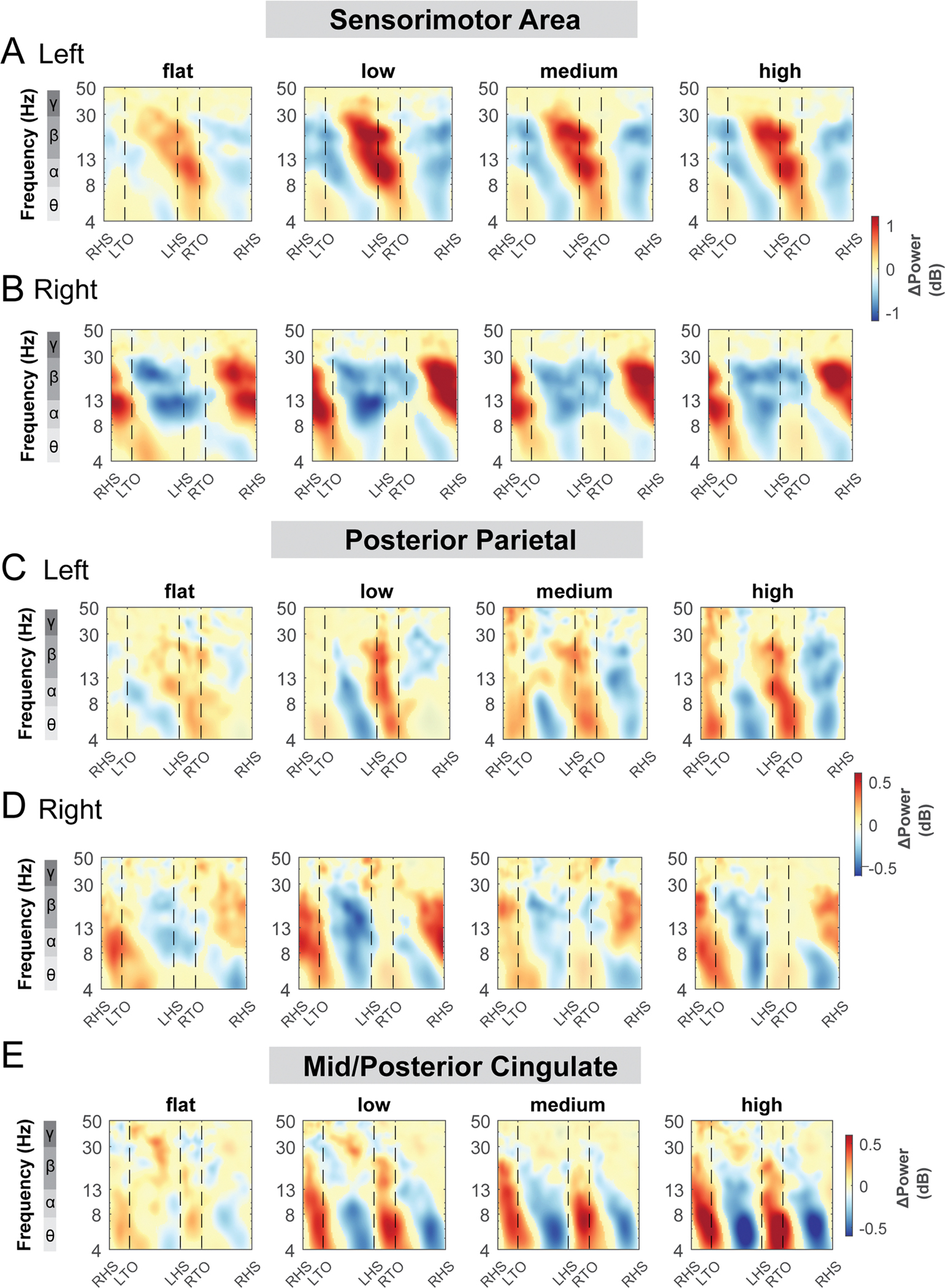
Event-related spectral perturbations at the sensorimotor (A - B), posterior parietal (C - D), and mid/posterior cingulate cluster (E) with respect to the average of each condition at different terrains. The x-axes of the ERSPs are time in gait cycle (RHS: right heel strike; LTO: left toe off; LHS: left heel strike; RTO: right toe off). All unmasked colors are statistically significant spectral power fluctuations relative to the mean power within the same condition. Colors indicate significant increases (red, synchronization) and decreases (blue, desynchronization) in spectral power from the average spectrum for all gait cycles to visualize intra-stride changes in the spectrograms. These data are significance masked (p < 0.05) through nonparametric bootstrapping with multiple comparison correction using false discovery rate.

Alpha band and beta band ERSPs showed lateralization for left and right sensorimotor clusters (Fig. 9A, B). Gait-related alpha and beta power desynchronization occurred during the contralateral limb swing phase while alpha and beta synchronization occurred during the contralateral limb stance phase and push-off.

There was also rhythmic ERSPs modulation across the gait cycle at both left and right posterior parietal clusters. For the left posterior parietal cluster, we observed theta and alpha power desynchronization during the swing phase and synchronization during the double support phase across all terrain conditions (Fig. 9C). As the terrain became more difficult, descriptively, we observed more broadband desynchronization during the contralateral swing phase. For the right posterior parietal cluster, we found broadband desynchronization during the swing phase in the low, medium, and high terrain conditions, as well as synchronization during the contralateral push-off phase (Fig. 9D).

ERSPs computed relative to the average power of the same condition at the mid/posterior cingulate areas visually increased with terrain unevenness (Fig. 9E). We found significant theta and alpha band synchronization during the double support phase and desynchronization following mid-swing when walking over uneven terrain (Fig. 9E).

We also performed similar analyses for the left pre-supplementary motor, right premotor, occipital, and caudate clusters (Supplementary Fig. 1). There was synchronization during the double support phase and desynchronization around the mid-swing in theta and alpha band for the left pre-supplementary motor (Supplementary Fig. 1A) and right premotor area (Supplementary Fig. 1B). The fluctuation became more prominent during uneven terrain walking than walking on the flat surface. At the occipital area, we observed broadband (theta, alpha, and beta) synchronization during the double support phase and desynchronization around the mid-swing (Supplementary Fig. 1C). At the caudate area, we observed theta, alpha synchronization during the double support phase and desynchronization around the mid-swing during uneven terrain walking (Supplementary Fig. 1D).

### 3.5. Effects of terrain unevenness on event-related power perturbations

**Fig. 10.**
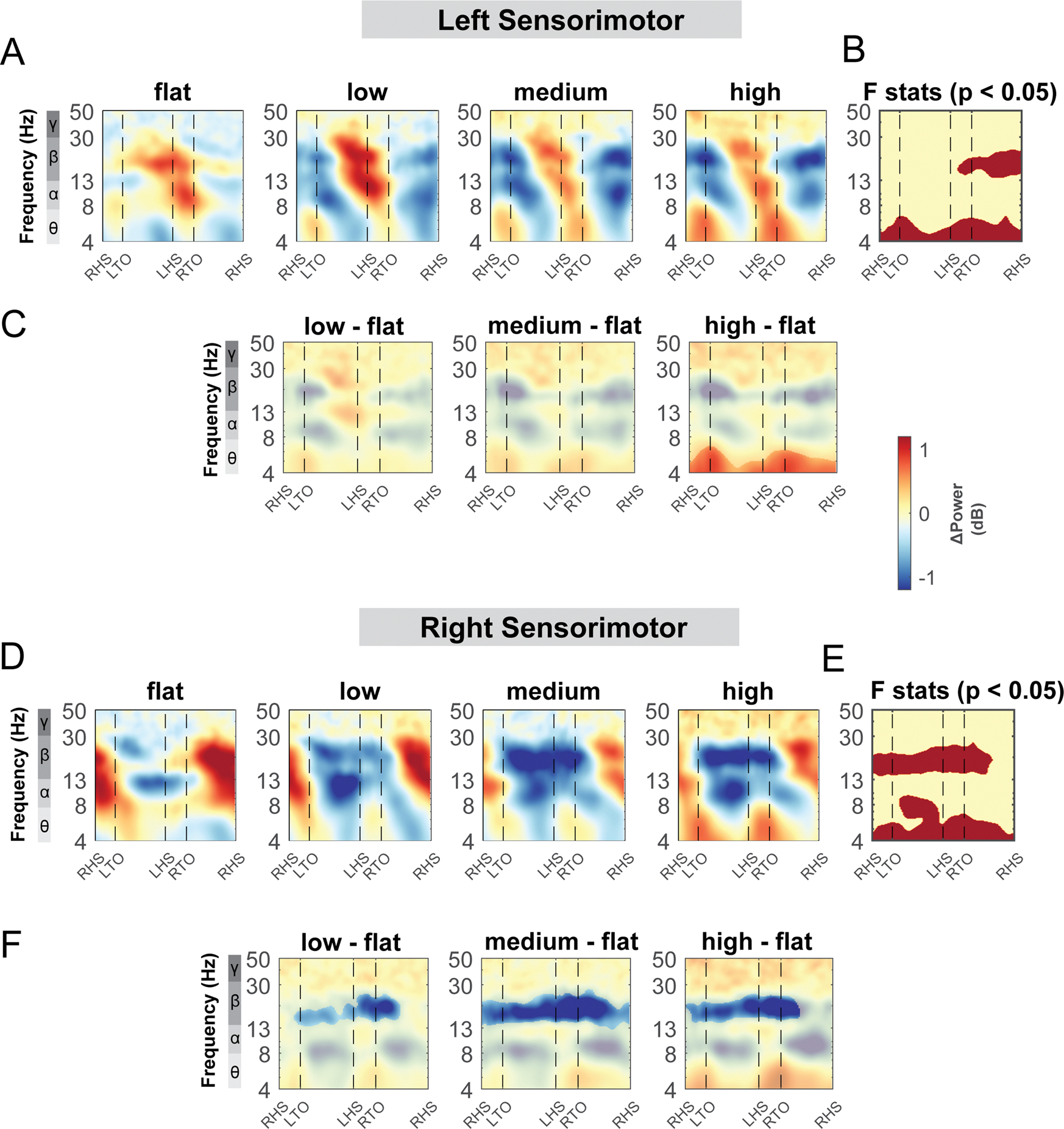
ERSPs at the left and right sensorimotor area with respect to the grand average of all conditions and with respect to the flat terrain condition. Averaged ERSP at different terrain at the left (A) and right sensorimotor cluster (D). Red indicated spectral power increase (neural synchronization) and blue indicated spectral power decrease (neural desynchronization) relative to the grand average of all conditions. Vertical dashed lines indicated gait events. RHS: right heel strike; LTO: left toe off; LHS: left heel strike; RTO: right toe off. (B, E) Significant effect of terrain on ERSPs across gait cycle with non-parametric statistics, with red indicating significance (p < 0.05). ERSPs with respect to flat condition at the left (C) and right (F) sensorimotor cluster. Regions that are not significantly different from flat condition have a semi-transparent mask.

We also computed the ERSPs with respect to the grand average of all conditions to assess the effect of terrain unevenness on spectral power fluctuation tied to gait events. All clusters showed spectral power fluctuation in event-related spectral perturbation plots at various frequency bands during the gait cycle with red indicating synchronization and blue indicating desynchronization (for example, Fig. 10A). We used non-parametric permutation statistics with cluster-based multiple comparison correction to determine the time-frequency differences across terrain conditions with red indicating significant differences across terrain conditions (p < 0.05; for example, Fig. 10B). To determine how spectral power changed relative to the flat condition, we computed the differences in ERSPs between each terrain condition relative to the flat condition (ERSP_terrain_ – ERSP_flat_) (for example, Fig. 10C). Regions that were not significantly different from the flat condition had a semi-transparent mask by using permutation statistics with cluster-based multiple comparison correction (for example, Fig. 10C).

#### 3.5.1. Sensorimotor areas

Theta, alpha, and beta power changed with terrain unevenness during distinct gait phases for both the left and right sensorimotor area (Fig. 10). At the left sensorimotor area, cluster-based permutation testing identified significant clusters in the theta band throughout the gait cycle and beta band during the contralateral swing phase (Fig. 10B, red region). There was greater theta power in the high terrain condition especially during the double support phase, compared to the flat condition while we did not find any significant differences in beta power using pairwise comparison (Fig. 10C).

At the right sensorimotor area, cluster-based permutation testing identified a significant cluster in the beta band during the double support phases and during the contralateral leg swing phase, with lower beta power in the low, medium, and high condition as compared to the flat condition (Fig. 10D – F). Lower beta power was observed following the contralateral foot strike to the ipsilateral swing in the low terrain condition as compared to the flat condition (Fig. 10F). Lower beta power was also observed during double support phases and the contralateral swing phase in the medium and high conditions. In addition, cluster-based permutation testing indicated a significant cluster in the alpha band from the contralateral mid-swing to the subsequent foot strike (Fig. 10E) although we did not find any significant pairwise difference with the flat terrain condition (Fig. 10F).

#### 3.5.2. Posterior parietal areas

**Fig. 11.**
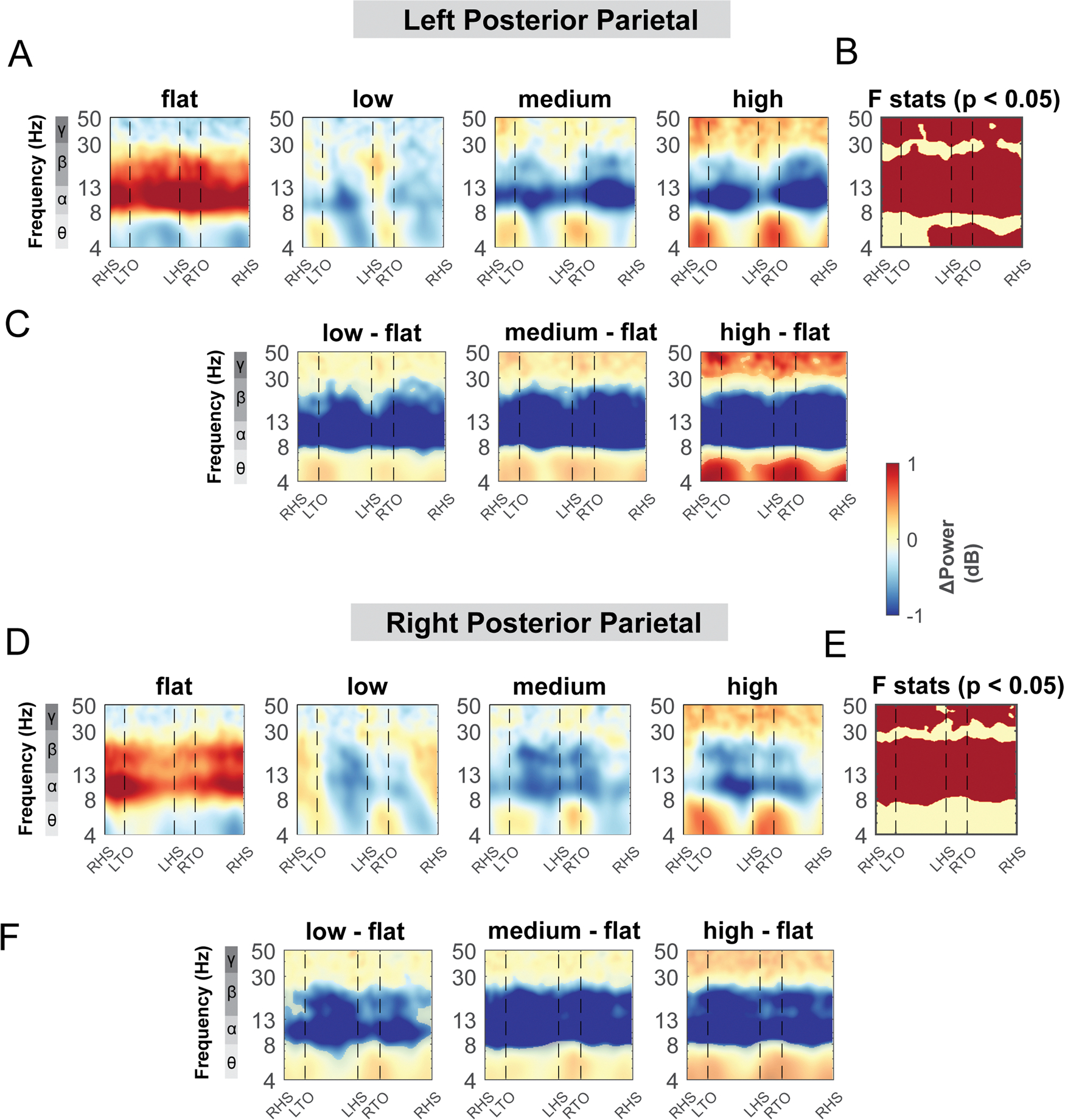
ERSPs at the left and right posterior parietal area with respect to the grand average of all conditions and with respect to the flat terrain condition. Averaged ERSP at different terrain at the left (A) and right posterior parietal cluster (D). Red indicated spectral power increase (neural synchronization) and blue indicated spectral power decrease (neural desynchronization) relative to the grand average of all conditions. Vertical dashed lines indicated gait events. RHS: right heel strike; LTO: left toe off; LHS: left heel strike; RTO: right toe off. (B, E) Significant effect of terrain on ERSPs across gait cycle with non-parametric statistics, with red indicating significance (p < 0.05). ERSPs with respect to flat condition at the left (C) and right (F) posterior parietal cluster. Regions that are not significantly different from flat condition have a semi-transparent mask as determined by cluster-based permutation.

Terrain modulated theta, alpha, and beta power across the gait cycle for both the left and right posterior parietal areas (Fig. 11). There was a significant cluster in the theta band across the gait cycle for the left posterior parietal area (Fig. 11B, red region), with greater theta power associated with greater terrain unevenness, especially at the double support phase. There was also a significant cluster in the alpha and beta band across the gait cycle for both posterior parietal areas, with lower alpha and beta power associated with greater terrain unevenness (Fig. 11B, E). Strong desynchronization at alpha and beta band was observed in both left and right clusters during all levels of uneven terrain walking as compared to that during flat terrain (Fig. 11C, F).

#### 3.5.3. Mid/posterior cingulate area

**Fig. 12.**
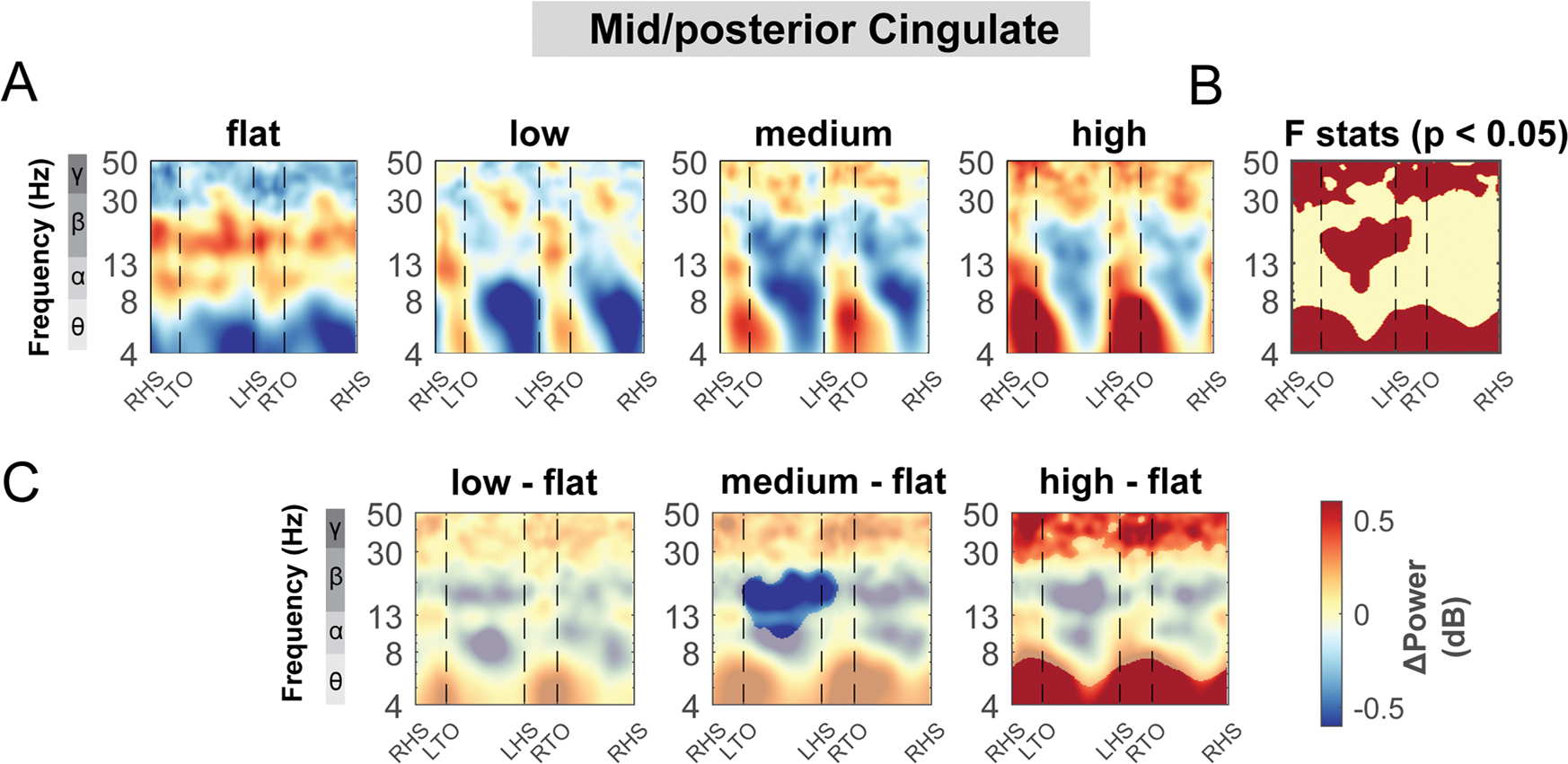
ERSPs at the mid/posterior cingulate area with respect to the grand average of all conditions and with respect to flat terrain condition. (A) Averaged ERSP at different terrain in the mid/posterior cingulate cluster. Red indicated spectral power increases (neural synchronization) and blue indicated spectral power decreases (neural desynchronization) relative to the grand average of all conditions. Vertical dashed lines indicated gait events. RHS: right heel strike; LTO: left toe off; LHS: left heel strike; RTO: right toe off. (B) Significant effect of terrain on ERSPs across gait cycle with non-parametric statistics, with red indicating significance (p < 0.05). (C) ERSPs with respect to flat condition at the mid/posterior cingulate cluster. Regions that are not significantly different from flat condition have a semi-transparent mask as determined by cluster-based permutation.

The mid/posterior cingulate area also demonstrated changes in theta, alpha, and beta band power during uneven terrain walking (Fig. 12). There was a significant cluster indicated by the cluster-based permutation testing in the theta power across the gait cycle, with greater theta power associated with greater terrain unevenness particularly at the double support phase (Fig. 12B). There was also a significant cluster in beta band during the left swing phase. We found lower beta power in the medium terrain condition compared to the flat terrain (Fig. 12C).

#### 3.5.4. Premotor, pre-supplementary motor, occipital, and caudate areas

The left premotor, right pre-supplementary motor, occipital, and caudate areas all showed power spectral fluctuation modulation by terrain (Supplementary Figs. 2 – 5). There was a significant cluster indicated by the cluster-based permutation testing in the theta and alpha band at the left premotor clusters (Supplementary Fig. 2B). Compared to the flat terrain, we found greater theta power in low, medium, and high terrain during the double support phase and the contralateral single-support stance phase at the left premotor cluster (Supplementary Fig. 2C). We also found greater alpha power in medium and high terrain conditions mainly during the double support phase (Supplementary Fig. 2C). At the right premotor cluster, there was a significant cluster in the theta band, with greater theta power observed in the high terrain condition compared to the flat terrain (Supplementary Fig. 3B, C). At the occipital cluster, we found significant clusters in the theta band power, with both greater theta power in at low, medium, and high terrain compared to flat condition (Supplementary Fig. 4). Additionally, at the caudate cluster, we found significant clusters in the theta and alpha (Supplementary Fig. 5). Greater theta and alpha power were observed at the double support phase in low and medium terrain condition and throughout the entire gait cycle at high terrain compared to the flat terrain.

### 3.6. Speed modulation of EEG power spectral density

#### 3.6.1. Sensorimotor areas

**Fig. 13.**
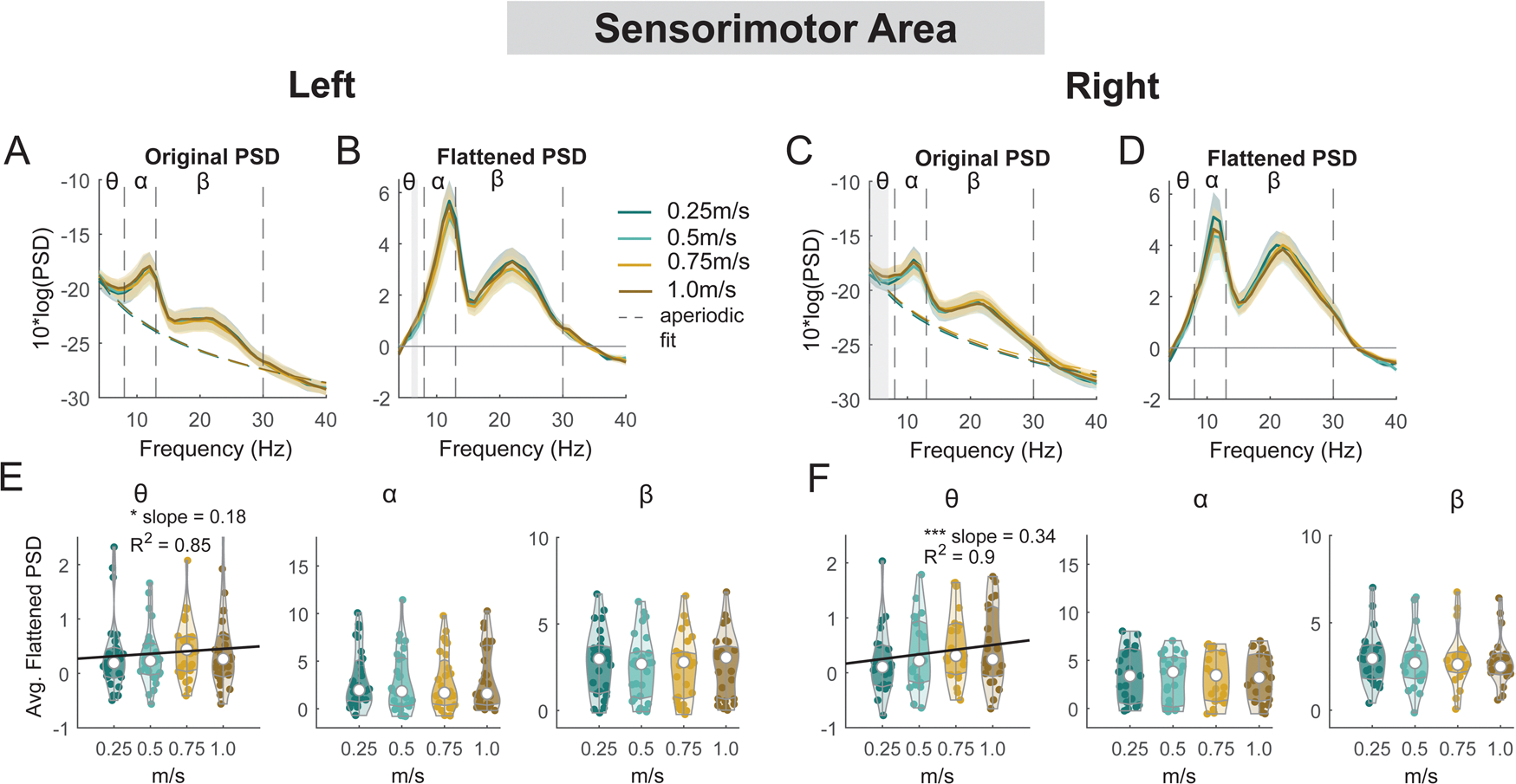
Power spectral density changes with walking speed at the left and right sensorimotor area. Average original PSDs changed with walking speed for the left (A) and right (C) sensorimotor cluster. Shaded colored areas indicated standard error of PSDs across components in the cluster. Dashed colored lines indicated average aperiodic fit. Gray shaded areas indicated a significant effect of terrain on PSDs. Vertical black dashed lines indicated main frequency bands of interest—theta (4 – 8 Hz), alpha (8 – 13 Hz), and beta (13 – 30 Hz). Average flattened PSDs after removing the aperiodic fit for the left (B) and right (D) sensorimotor cluster. (E - F) Average power for theta, alpha, and beta band computed from flattened PSDs for all components within the cluster for the left and right clusters. Black line indicates a significant correlation between average power and walking speed (*p < 0.05, ***p < 0.001).

**Fig. 14.**
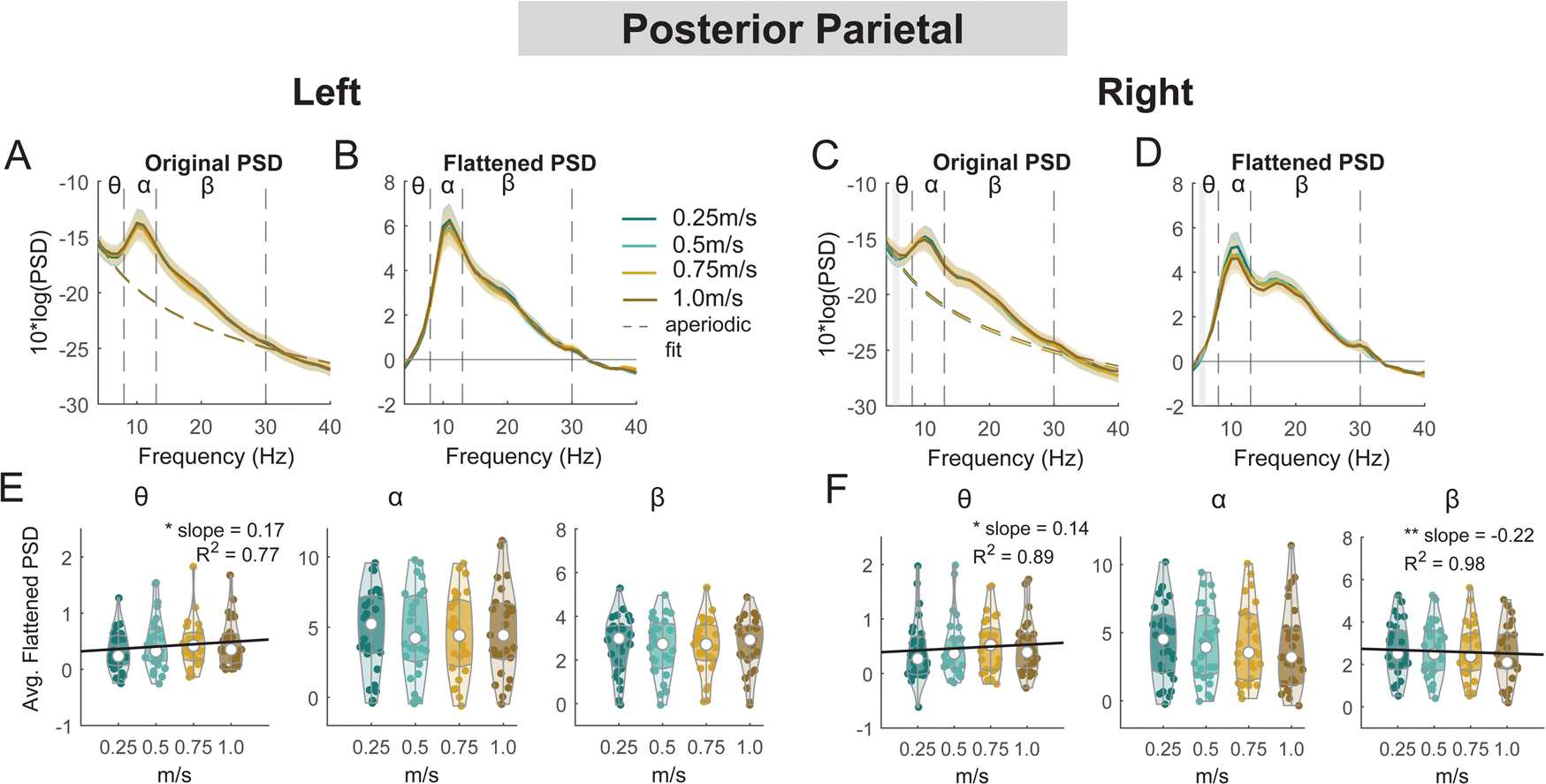
Power spectral density changes with walking speed at the left and right posterior parietal area. Average original PSDs changed with walking speed for the left (A) and right (C) posterior parietal cluster. Shaded colored areas indicated standard error of PSDs across components in the cluster. Dashed colored lines indicated average aperiodic fit. Gray shaded areas indicated a significant effect of terrain on PSDs. Vertical black dashed lines indicated main frequency bands of interest—theta (4 – 8 Hz), alpha (8 – 13 Hz), and beta (13 – 30 Hz). Average flattened PSDs after removing the aperiodic fit for the left (B) and right (D) posterior parietal cluster. (E - F) Average power for theta, alpha, and beta band computed from flattened PSDs for all components within the cluster for the left and right clusters. Black line indicates a significant correlation between average power and walking speed (*p < 0.05, **p < 0.01).

**Fig. 15.**
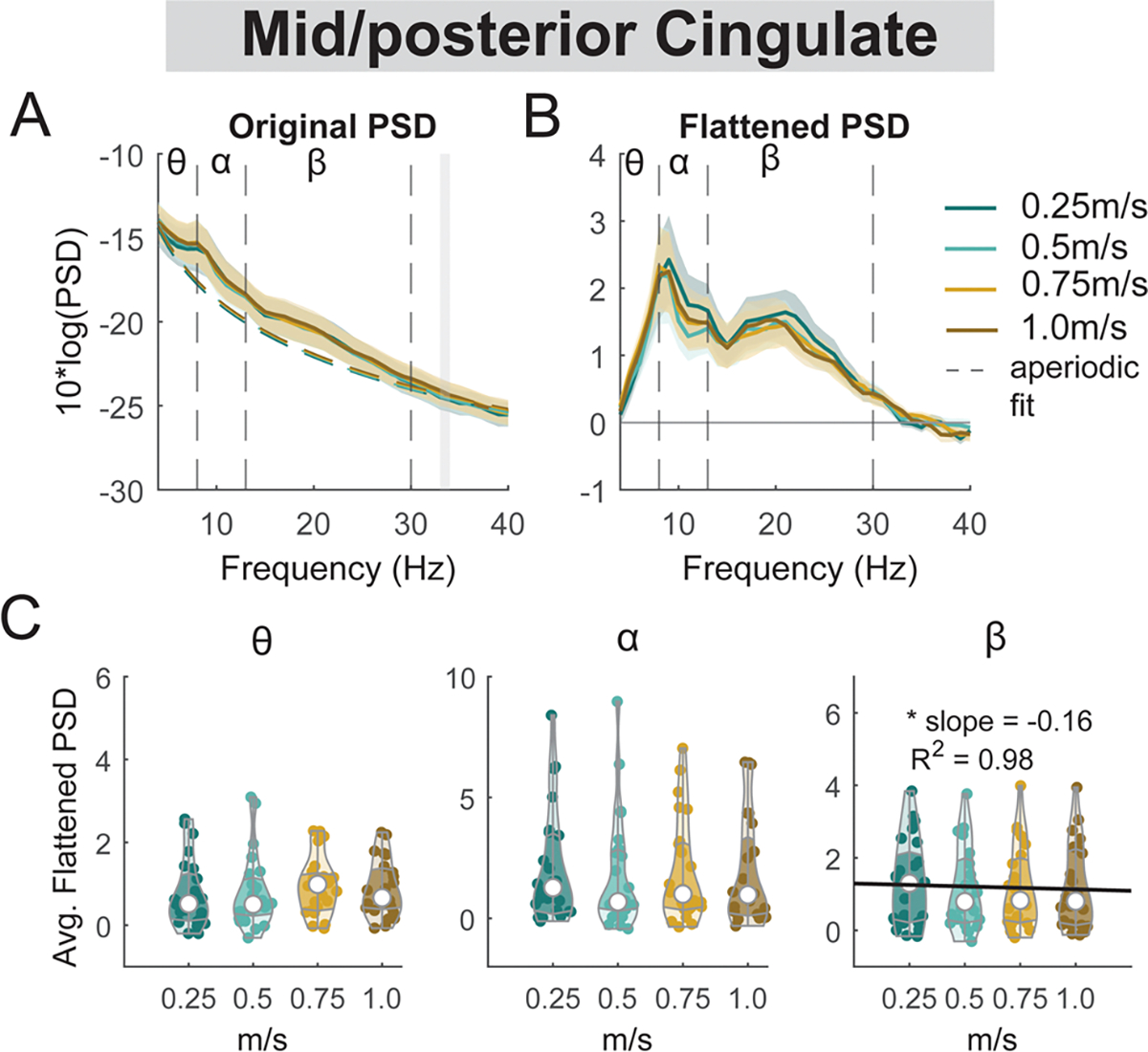
Power spectral density changes with walking speed at the mid/posterior cingulate area. (A) Average original PSDs changed with walking speed for the mid/posterior cingulate cluster. Shaded colored areas indicated standard error of PSDs across components in the cluster. Dashed colored lines indicated average aperiodic fit. Gray shaded areas indicated a significant effect of terrain on PSDs. Vertical black dashed lines indicated main frequency bands of interest—theta (4 – 8 Hz), alpha (8 – 13 Hz), and beta (13 – 30 Hz). (B) Average flattened PSDs after removing the aperiodic fit for the mid/posterior cingulate cluster. (C) Average power for theta, alpha, and beta band computed from flattened PSDs for all components within the cluster. Black line indicates a significant correlation between average power and walking speed (*p < 0.05).

For the secondary objective of this study, we employed a linear mixed-effect model for each outcome measure (average power for each band for each cluster) to assess the effect of walking speed on EEG spectral power at multiple cortical areas (Figs. 13 – 15). There was a main effect of speed on average theta power (F(1, 110) = 4.2, p = 0.04, Cohen’s f^2^ = 0.055) at the left sensorimotor area, with greater theta power associated with faster walking speed (Fig. 13E). However, we did not find a significant main effect of speed on alpha power (F(1, 110) = 0.5, p = 0.5, Cohen’s f^2^ = 0.006) or beta power (F(1, 110) = 1.6, p = 0.2, Cohen’s f^2^ = 0.0192). Additionally, we observed a main effect of speed on average theta power at the right sensorimotor cluster (F(1, 82) = 14.9, p < 0.001, Cohen’s f^2^ = 0.26), with greater theta power at faster walking speed. There was no effect of speed on alpha power (F(1, 82) = 0.39, p = 0.5, Cohen’s f^2^ = 0.0064) or beta power (F(1, 82) = 2.1, p = 0.15, Cohen’s f^2^ = 0.035).

#### 3.6.2. Posterior parietal areas

For the left posterior parietal cluster, there was a main effect of speed on average theta power (F(1, 106) = 5.3, p = 0.023, Cohen’s f^2^ = 0.076), with greater theta power associated with faster walking speed (Fig. 14C). However, we did not find a significant main effect of speed on alpha power (F(1, 106) = 0.04, p = 0.83, Cohen’s f^2^ = 0) or beta power (F(1, 106) = 0.22, p = 0.64, Cohen’s f^**2**^ = 0.003). For the right posterior parietal cluster, we found a main effect of speed on average theta power (F(1, 110) = 4.2, p = 0.04, Cohen’s f^2^ = 0.055), with greater theta power associated with faster walking speed. We also found a main effect of speed on average beta power (F(1, 110) = 8.4, p = 0.005, Cohen’s f^2^ = 0.1) with lower beta power associated with faster walking speed. There was no effect of speed on alpha power (F(1, 110) = 3.85, p = 0.052, Cohen’s f^2^ = 0.048).

#### 3.6.3. Mid/posterior cingulate area

There was a main effect of speed on the average beta power (F(1, 94) = 5.4, p = 0.023, Cohen’s f^2^ = 0.047) at the mid/posterior cingulate, with lower beta power associated with faster walking speed (Fig. 15). However, we did not find a significant main effect of speed on theta power (F(1, 94) = 3.2, p = 0.08, Cohen’s f^2^ = 0.01) or alpha power (F(1, 94) = 0.65, p = 0.42, Cohen’s f^2^ = 0.076).

#### 3.6.4. Premotor, pre-supplementary motor, occipital, and caudate areas

**Fig. 16.**
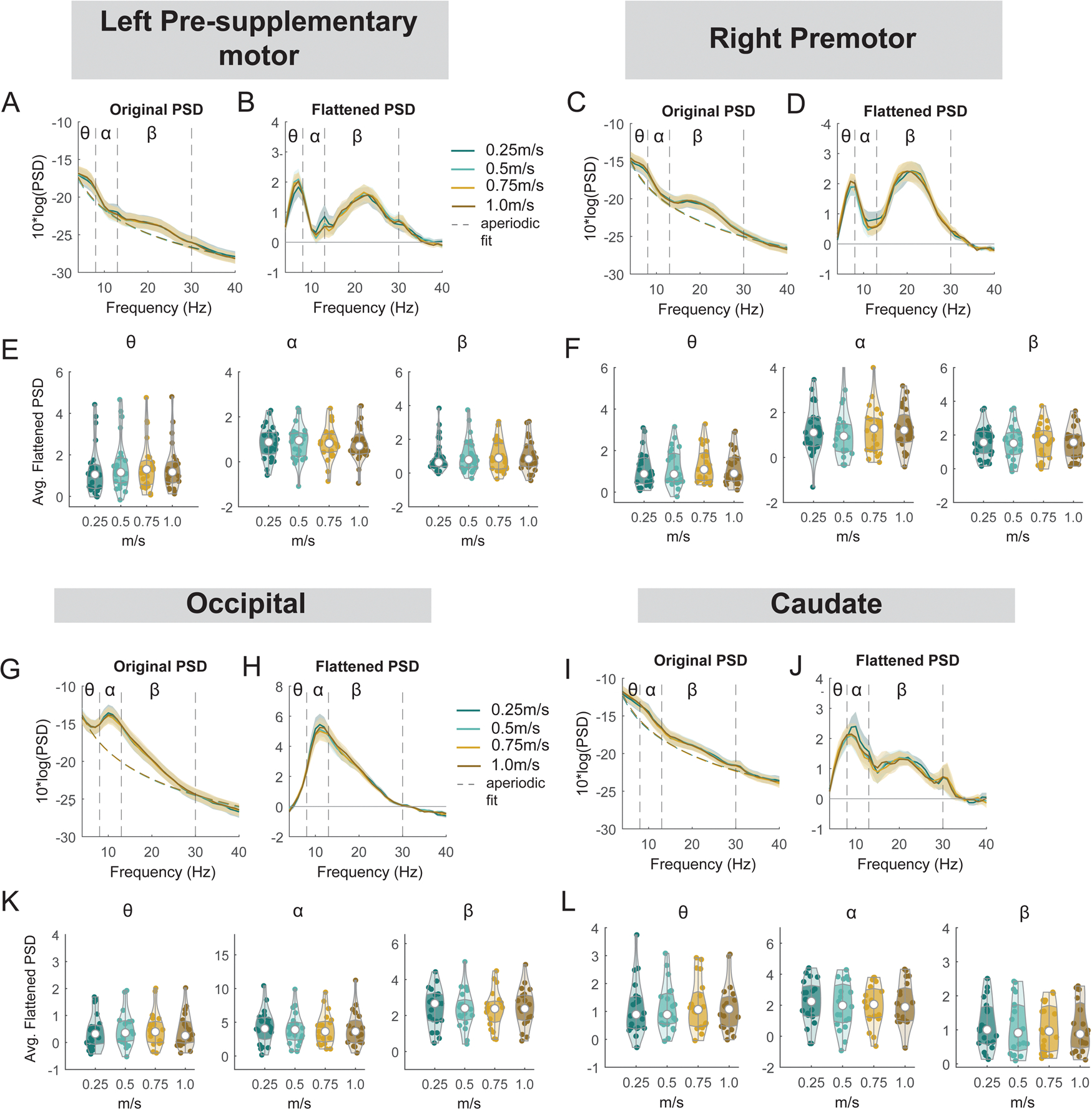
Power spectral density changes with walking speed at the left pre-supplementary motor, right premotor, occipital, and caudate area. (A) Average original PSDs changed with walking speed for the left pre-supplementary cluster. Shaded colored areas indicated standard error of PSDs across components in the cluster. Dashed colored lines indicated average aperiodic fit. Gray shaded areas indicated a significant effect of speed on PSDs. Vertical black dashed lines indicated main frequency bands of interest—theta (4 – 8 Hz), alpha (8 – 13 Hz), and beta (13 – 30 Hz). (B) Average flattened PSDs after removing the aperiodic fit for the left pre-supplementary motor cluster. (E) Average power for theta, alpha, and beta band computed from flattened PSDs for all components within the cluster for the left pre-supplementary clusters. Black line indicates a significant correlation between average power and walking speed. (C) Average original PSDs changed with walking speed for the right premotor cluster. (D) Average flattened PSDs after removing the aperiodic fit for the right premotor cluster. (F) Average power for theta, alpha, and beta band computed from flattened PSDs for all components within the right premotor cluster. (G) Average original PSDs changed with walking speed for the occipital cluster. (H) Average flattened PSDs after removing the aperiodic fit for the occipital cluster. (K) Average power for theta, alpha, and beta band computed from flattened PSDs for all components within the occipital cluster. (I) Average original PSDs changed with walking speed for the caudate cluster. (J) Average flattened PSDs after removing the aperiodic fit for the caudate cluster. (L) Average power for theta, alpha, and beta band computed from flattened PSDs for all components within the caudate cluster.

Similarly, we performed an exploratory analysis on the effect of walking speed on band powers at the left pre-supplementary motor, right premotor, occipital, and caudate clusters (Fig. 16). There was no effect of speed on theta, alpha, or beta power at either left pre-supplementary motor, right premotor cluster, occipital, or caudate cluster (all p > 0.05).

### 3.7. Gait-related spectral perturbations during walking at different speeds

We again computed the event-related power perturbations within the gait cycle at each walking speed with respect to the average power across the gait cycle of the same condition at each frequency (Gwin et al., 2011). We unmasked the significant deviations from the average spectrum of each condition with a bootstrap method with false discovery rate multiple comparison correction.

**Fig. 17.**
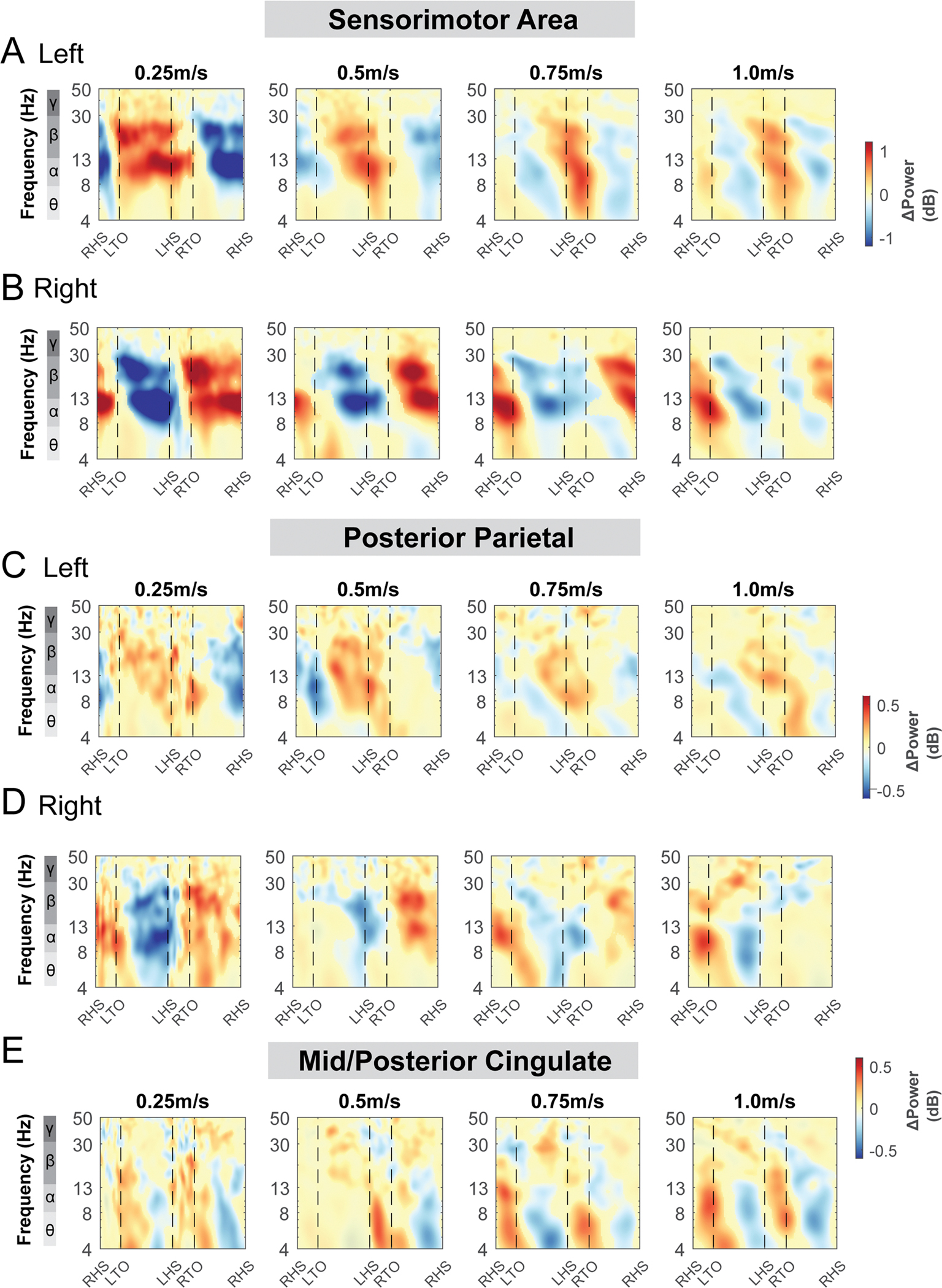
ERSPs at the sensorimotor (A - B), posterior parietal (C - D), and mid/posterior cingulate areas (E) with respect to the average of each condition at different speeds. The x-axes of the ERSPs are time in gait cycle (RHS: right heel strike; LTO: left toe off; LHS: left heel strike; RTO: right toe off). All unmasked colors are statistically significant spectral power fluctuations relative to the mean power within the same condition. Colors indicate significant increases (red, synchronization) and decreases (blue, desynchronization) in spectral power from the average spectrum for all gait cycles to visualize intra-stride changes in the spectrograms. These data are significance masked (p < 0.05) through nonparametric bootstrapping with multiple comparison correction using false discovery rate.

Similar to Figure 9, alpha band and beta band activity showed lateralization for left and right sensorimotor clusters at different walking speeds (Fig. 17A, B). We observed alpha and beta desynchronization during the contralateral swing phase and synchronization during the ipsilateral swing phase at 0.25 m/s and 0.5 m/s. At higher walking speeds (0.75 m/s and 1.0 m/s), we observed additional alpha and beta synchronization during the double support phase from the ipsilateral foot strike until the contralateral foot off. Descriptively, power fluctuations decreased with faster walking speed at the alpha and beta band.

At the posterior parietal area, the spectral power fluctuations across the gait cycle were not consistent across the speeds (Fig. 17C, D). We observed alpha and beta desynchronization during the contralateral swing phase and synchronization during the contralateral swing phase at slower walking speeds (0.25 m/s and 0.5 m/s). At the mid/posterior cingulate cluster, we observed theta and alpha synchronization during both double support phase and desynchronization during swing phase (Fig. 17E). At the supplementary motor clusters, occipital cluster, and caudate clusters, power spectral fluctuation within each condition was not different across speeds (Supplementary Fig. 6). Only at higher speeds (0.75 m/s and 1.0 m/s) did we observe prominent theta and alpha band synchronization during the double support phase and desynchronization at mid-swing phase at the supplementary motor, occipital, and caudate clusters.

### 3.8. Effects of walking speed on event-related power perturbations

**Fig. 18.**
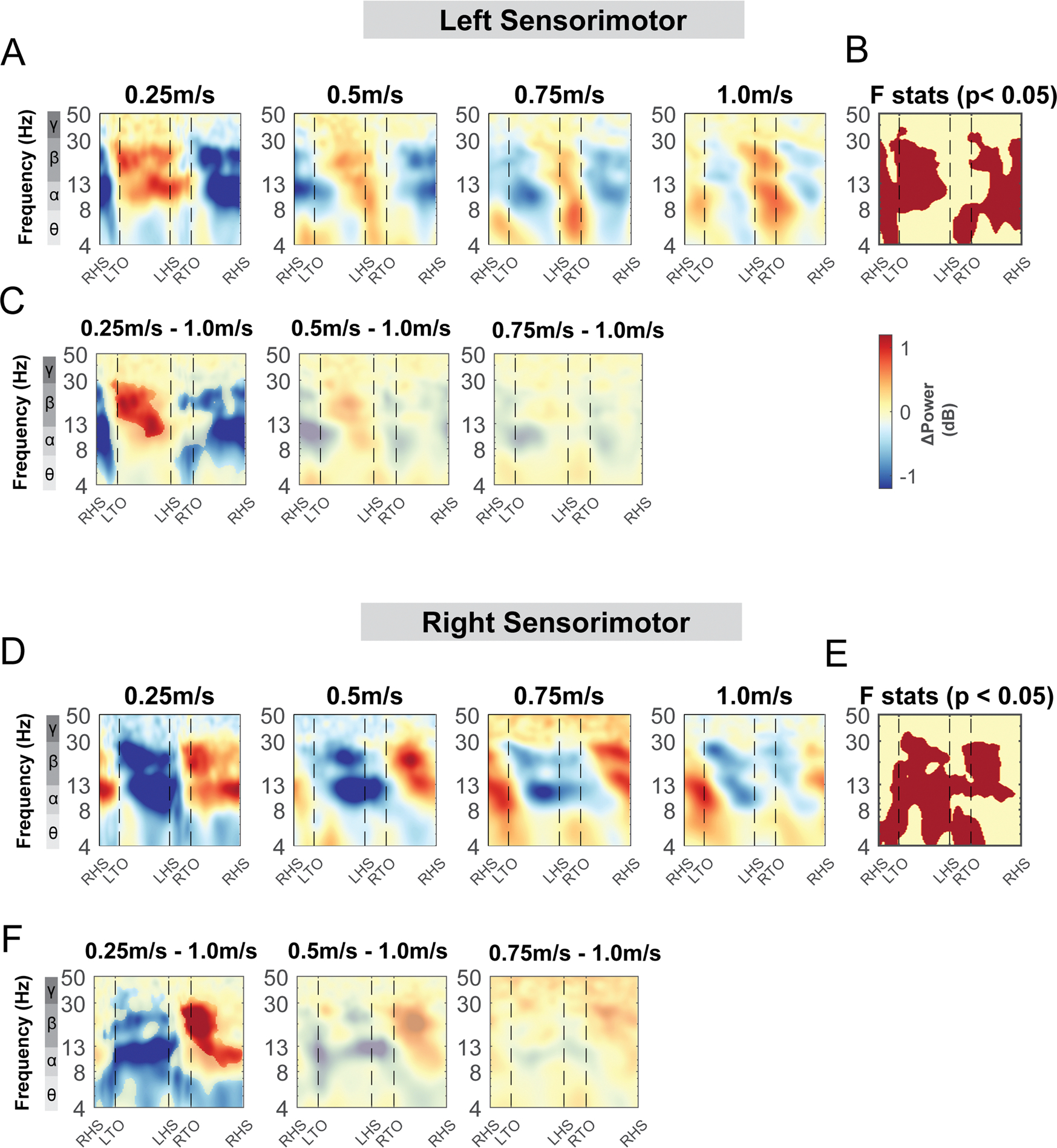
ERSPs at the sensorimotor area with respect to the grand average of all conditions and with respect to the 1.0 m/s speed condition. Averaged ERSP at different speeds at the left (A) and right sensorimotor cluster (D). Red indicated spectral power increases (neural synchronization) and blue indicated spectral power decreases (neural desynchronization) relative to the grand average of all conditions. Vertical dashed lines indicated gait events. RHS: right heel strike; LTO: left toe off; LHS: left heel strike; RTO: right toe off. (B - E) Significant effect of terrain on ERSPs across gait cycle with non-parametric statistics, with red indicating significance (p < 0.05). ERSPs with respect to 1.0 m/s speed condition at the left (C) and right (F) sensorimotor cluster. Regions that are not significantly different from 1.0 m/s condition have a semi-transparent mask.

We then computed the ERSPs with respect to the grand average of all conditions to assess the effect of walking speed on spectral power fluctuation tied to gait events. All clusters showed spectral power fluctuation in event-related spectral perturbation plots at various frequency bands during the gait cycle with red indicating synchronization and blue indicating desynchronization (for example, Fig. 18A). We used non-parametric permutation statistics with cluster-based multiple comparison correction to determine the time-frequency differences across speed conditions with red indicating significant differences across speed conditions (p < 0.05; for example, Fig. 18B). To determine how spectral power changed relative to the 1.0 m/s condition, we computed the differences in ERSPs between each speed condition relative to the 1.0 m/s condition (ERSP_terrain_ – ERSP_1.0 m/s_) (for example, Fig. 18C). Regions that were not significantly different from 1.0 m/s condition have a semi-transparent mask using permutation statistics with cluster-based multiple comparison correction (for example, Fig. 18C).

#### 3.8.1. Sensorimotor areas

Theta, alpha, and beta power fluctuations changed with walking speed for both the left and right sensorimotor areas (Fig. 18A, D). We found a significant cluster indicated by cluster-based permutation testing in the theta band during double support phases for both left and right sensorimotor areas (Fig. 18B, E). Theta power was lower during the double support phase when walking at 0.25 m/s compared to 1.0 m/s. There was also a significant cluster in the theta band during the contralateral swing phase in both the left and right sensorimotor areas. We observed a lower theta power when walking at 0.25 m/s compared to 1.0 m/s (Fig. 18C, F). Additionally, we observed a significant cluster in the alpha band during the swing phase at both left and right sensorimotor areas. We found a lower alpha power at 0.25 m/s during the contralateral swing phase and a greater alpha power during the contralateral stance phase at 0.25 m/s compared to 1.0 m/s. Lastly, we found a significant cluster in the beta band during both swing phases (Fig. 18B, E). Beta power was greater during the contralateral swing phase and lower during the contralateral stance phase when walking at 0.25 m/s compared to 1.0 m/s.

#### 3.8.2. Posterior parietal clusters, mid/posterior cingulate clusters, and other areas

**Fig. 19.**
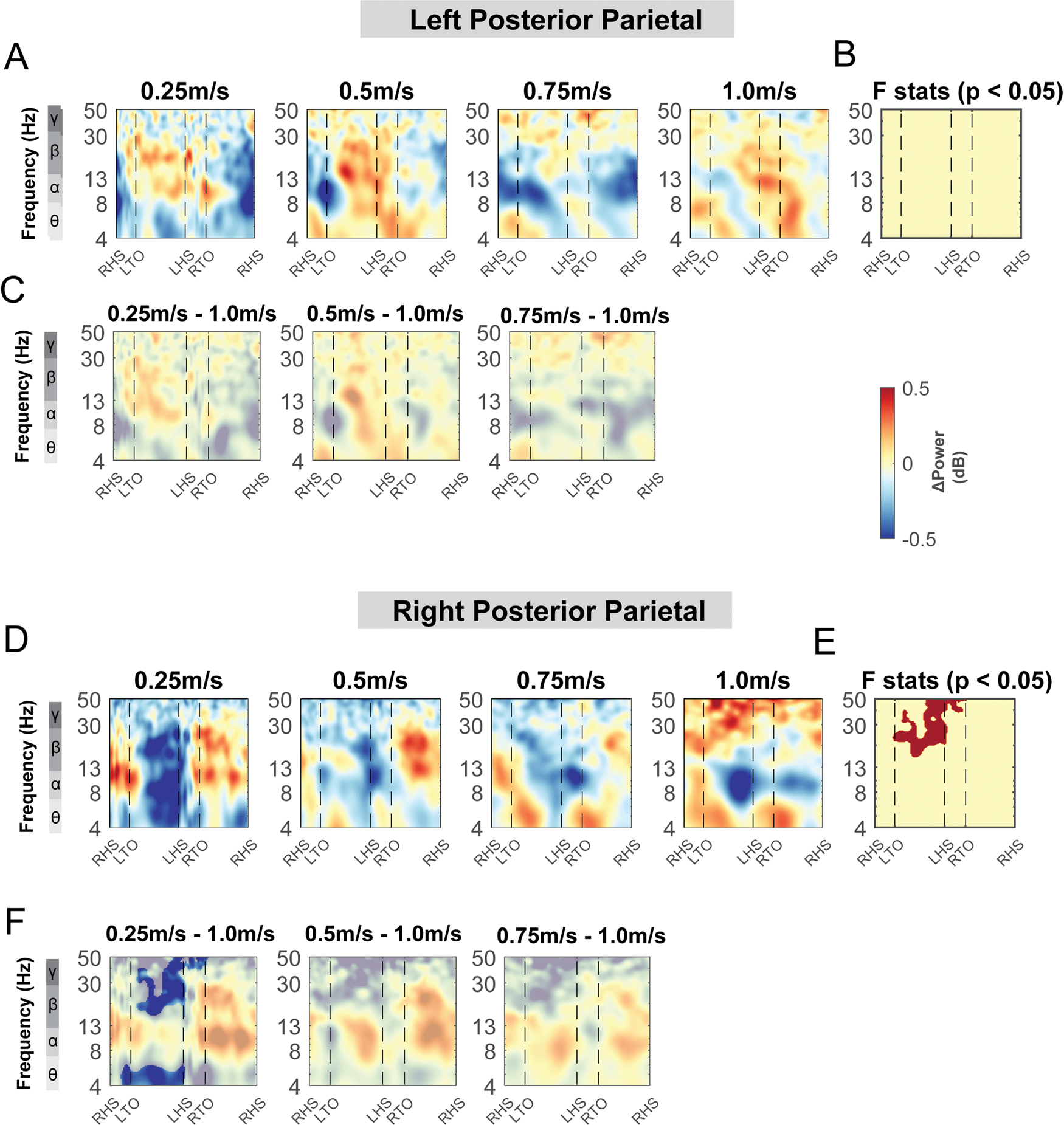
ERSPs at the posterior parietal area with respect to the grand average of all conditions and with respect to the 1.0 m/s speed condition. Averaged ERSP at different speeds at the left (A) and right posterior parietal cluster (D). Red indicated spectral power increases (neural synchronization) and blue indicated spectral power decreases (neural desynchronization) relative to the grand average of all conditions. Vertical dashed lines indicated gait events. RHS: right foot strike; LTO: left foot off; LHS: left foot strike; RTO: right foot off. (B, E) Significant effect of terrain on ERSPs across gait cycle with non-parametric statistics, with red indicating significance (p < 0.05). ERSPs with respect to 1.0 m/s speed condition at the left (C) and right (F) posterior parietal cluster. Regions that are not significantly different from 1.0 m/s condition have a semi-transparent mask.

**Fig. 20.**
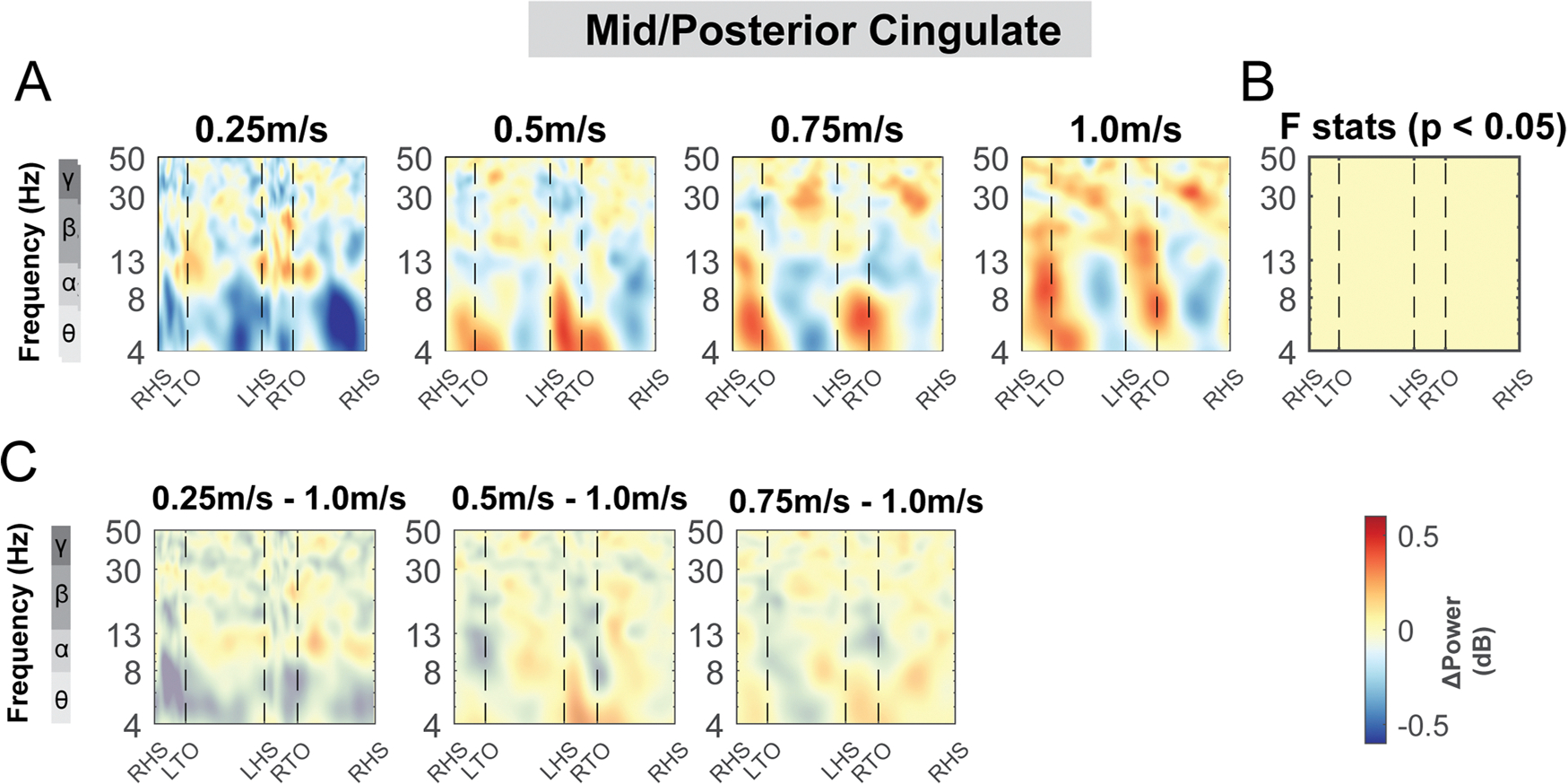
ERSPs at the mid/posterior cingulate area with respect to the grand average of all conditions and with respect to the 1.0 m/s speed condition. Averaged ERSP at different speeds at mid/posterior cingulate cluster (A). Red indicated spectral power increases (neural synchronization) and blue indicated spectral power decreases (neural desynchronization) relative to the grand average of all conditions. Vertical dashed lines indicated gait events. RHS: right heel strike; LTO: left toe off; LHS: left heel strike; RTO: right toe off. (B) Significant effect of terrain on ERSPs across gait cycle with non-parametric statistics, with red indicating significance (p < 0.05). (C) ERSPs with respect to 1.0 m/s speed condition. Regions that are not significantly different from 1.0 m/s condition have a semi-transparent mask.

We did not find any effect of speed on ERSPs at the posterior parietal areas, except for beta power during the contralateral swing phase at the right posterior parietal clusters (Fig. 19B, E). We found a lower beta power during the contralateral swing phase at the right posterior parietal clusters when walking at 0.25 m/s versus 1.0 m/s (Fig. 19E, F). We also did not find any effect of speed on ERSPs at the mid/posterior cingulate area (p > 0.05; Fig. 20).

There was an effect of speed on ERSPs in theta band during the double support phase only for the left pre-supplementary motor cluster (p < 0.05) while we did not find any differences in ERSPs using pairwise comparison referenced to the 1.0 m/s condition (Supplementary Fig. 7). For other clusters including the right premotor, occipital, and caudate clusters, we did not find any significant effect of speed on ERSPs (all p’s > 0.05) (Supplementary Figs. 8 – 10).

## 4. DISCUSSION

Our study’s primary objective was to determine how electrocortical activity measured by EEG changed with parametric variations in terrain unevenness for neurotypical young adults. We identified multiple brain regions associated with uneven terrain walking. We found that alpha and beta spectral power were lower with greater terrain unevenness at the sensorimotor and posterior parietal areas while theta spectral power was greater in the mid/posterior cingulate area with greater terrain unevenness. We also observed that gait-related spectral power fluctuations changed with terrain unevenness in all identified brain clusters. Our secondary goal was to determine how electrocortical activity changed with walking speed. Contrary to our speed hypothesis, we found that alpha and beta average spectral power did not change with walking speed in the sensorimotor areas. We only observed a significant effect of speed on gait-related spectral power fluctuations in the sensorimotor area but not much in other brain areas. These results suggest that distinct cortical processes may be recruited for walking over uneven terrain versus flat terrain at different speeds. This also confirms that the observed cortical changes during uneven terrain walking were not related to the variability in walking speed between participants. Slower gait speed and difficulty in traversing uneven terrain are both characteristics of mobility impairment as humans get older as well as in other conditions known to impact mobility (e.g., chronic pain, post-stroke hemiparesis, lower limb amputation) (Downey et al., 2022; Ogawa et al., 2020; Von Schroeder et al., 1995). Providing this baseline data of electrocortical dynamics in neurotypical young participants would help better understand cortical deficiencies that occur across the lifespan and lead to a better understanding of neural compensation for future studies (Clark et al., 2020; Fettrow et al., 2021). Our results could also help reinforce the importance of cortical involvement in the control of human walking, often characterized as primarily dependent on reflex activation and spinal neural networks.

### 4.1. Alpha and beta power decrease with terrain unevenness

Consistent with our hypothesis, alpha and beta spectral power across the gait cycle were lower with greater terrain unevenness at the sensorimotor area. Alpha oscillations are considered to reflect an “idling” state of the brain (Mulholland, 1995) and lower alpha band power indicates active cortical processing. In the sensorimotor region, the alpha and beta band power decrease following movement initiation is attributed to an increased cortical neuron activity for motor planning and execution (Deiber et al., 2012; Pfurtscheller & Lopes da Silva, 1999). Our results suggest that cortical involvement was greater during uneven terrain walking versus walking on flat surfaces. Beta power reduction during uneven terrain walking was prominent during the contralateral limb swing phase before foot placement in our study, which may be indicative of goal-directed visuomotor processing prior to foot placement during uneven terrain walking. Our result is consistent with other studies involving goal-directed movements (Studnicki & Ferris, 2023; Tombini et al., 2009). For example, beta desynchronization was observed before hitting a table tennis ball (Studnicki & Ferris, 2023) or intercepting an object on the screen (Tombini et al., 2009) compared to premovement.

However, there was some discrepancy between our finding of beta desynchronization in sensorimotor area and that in a previous study by Jacobsen et al. (2022). Jacobsen et al. found beta power reduction only following foot strike (Jacobsen et al., 2022). Several factors may contribute to the discrepancy. The terrain was more challenging in our study compared to that in Jacobsen et al. as they compared paved overground concrete terrain with unpaved grassy terrain. It seems probable that walking on our uneven terrain treadmill required more cortical processing regarding movement adjustments during swing compared to walking on the unpaved grassy terrain. Another reason could be that we used a clustering approach to group the brain components based on each source’s location before computing the average spectral power across participants. In contrast, Jacobsen et al. computed the spectral power modulation in the channel-space at the Cz electrode, which limits their interpretation of the location of the sources. As a result, beta power reduction at Cz electrode following right foot strike may have contributions from other non-sensorimotor areas.

The posterior parietal area demonstrated sustained alpha and beta power desynchronization across the gait cycle during uneven terrain walking compared to walking on a flat surface (Fig. 11). The posterior parietal area is associated with multisensory integration and estimation of an obstacle’s location relative to the body’s current state to appropriately modify the gait pattern (Drew & Marigold, 2015; Marigold & Drew, 2017). Lower alpha and beta power may reflect cortical processing of multisensory modalities (i.e., vision, vestibular, and proprioception) to maintain balance when walking on an uneven terrain. In addition, a sustained decrease in alpha power across the gait cycle in the posterior parietal area could be attributed to greater attention to balance control and greater alertness to threat perception during uneven terrain walking compared to walking on an even surface (Sarter et al., 2001). Greater attention can help prioritize task-relevant sensory processing during gait to filter task-irrelevant stimuli or noise (Sarter et al., 2001). Therefore, alpha power change at the posterior parietal area can potentially be used as a cortical marker of sensorimotor attention and alertness during gait for future studies that investigate sustained attention and its relationship with mobility deficits.

### 4.2. Theta band power increase with terrain unevenness

Inconsistent with our hypothesis, we did not find a cluster at the anterior cingulate area but rather at the mid/posterior cingulate area, which plays a role in somatosensory processing (Seitz & Roland, 1992) and orientation of the body in space to sensory stimuli (Vogt, 2016). Multiple other EEG studies have reported mid/posterior cingulate involvement during locomotor tasks that required balance control. Sipp et al. identified a posterior cingulate cluster during a narrow beam walking task in which theta band power significantly increased following loss of balance (Sipp et al., 2013). In a different study, participants walked in a split-belt environment where one belt speed moved faster than the other. Both anterior and posterior cingulate clusters showed strong theta synchronization during early adaptation (when balance was challenged) versus pre-adaptation (when participants walked with tied-belt speed) (Jacobsen & Ferris, 2023). It is likely that the mid/posterior cingulate area receives multi-sensory input from sensorimotor cortex and parietal cortex to guide body orientation and movements during balance challenging tasks (Cavanagh & Frank, 2014; Vogt, 2016). Future studies should investigate the effective connectivity between the mid/posterior cingulate and sensorimotor area and posterior parietal to better determine the role of the mid/posterior cingulate in maintaining balance during gait.

On more uneven terrain, greater theta band power was slightly associated with greater terrain unevenness in the mid/posterior cingulate cluster (Fig. 7). Additionally, we found theta synchronization across the gait cycle during the most challenging terrain condition compared to the flat condition in the left sensorimotor, left posterior parietal, mid/posterior cingulate, left pre-supplementary motor, right premotor, and occipital cluster. These results indicated that theta synchronization is widely distributed across the brain, and a higher level cognitive control is needed during the more complex locomotor task (Cavanagh & Frank, 2014; Raghavachari et al., 2006). Additionally, theta oscillation may facilitate multisensory integration during movement. For example, theta power was higher in a congenitally blind participant compared to normally sighted participants when walking freely in a room, indicating that theta power may be associated with somatosensory processing during movement (Aghajan et al., 2017). Together, greater theta power when walking on a more uneven surface may be attributed to a greater need for sensory processing to maintain balance during uneven terrain walking (Raghavachari et al., 2006).

### 4.3. Gait-related spectral power fluctuations

Patterns of event-related spectral power fluctuations in the sensorimotor area are in line with previous literature using both non-invasive and invasive recordings (Bradford et al., 2016; McCrimmon et al., 2018; Nordin et al., 2020; Oliveira et al., 2017; Zhao et al., 2022). The power spectral fluctuations computed with respect to the average spectral power within each condition showed significant alpha and beta desynchronization during the contralateral swing phase and synchronization during the contralateral limb stance phase and push-off (Fig. 9). The spectral power fluctuation profile at the sensorimotor area found in this study is consistent with the neural activation profiles classically recorded in rats, rabbits, cats, and nonhuman primates (Armstrong & Drew, 1984; Beloozerova & Sirota, 1993; DiGiovanna et al., 2016). Neuron firing rates peaked during the gait phase transition and swing phase in rats, and likewise, cortical motor neurons in cats and primates also demonstrated increased firing rates toward push-off phase and swing phase (Armstrong & Drew, 1984; Beloozerova & Sirota, 1993; DiGiovanna et al., 2016). These results suggested increased cognitive processing for movement planning during the swing phase.

We also observed gait-related spectral power fluctuations at the non-sensorimotor areas when participants walked on both flat surfaces and uneven terrain. For instance, there was rhythmic modulation of power spectral fluctuations at the left and right posterior parietal clusters during walking on a flat surface (Fig. 9). Such modulation is similar to that observed in cats (Andujar et al., 2010; Beloozerova & Sirota, 2003) where neural population peak activity occurred during the swing phase of the contralateral forelimb at the area 5 of the posterior parietal cluster (Andujar et al., 2010; Beloozerova & Sirota, 2003). These results suggested that other brain areas may receive movement-related information from the sensorimotor area during locomotion. However, we also observed some differences between gait-related spectral fluctuations between the non-sensorimotor areas and sensorimotor areas. One difference is that we did not observe strong lateralization at the non-sensorimotor areas. This is likely because fewer limb-dependent cells exist at higher-level brain centers compared to the motor cortex (Andujar et al., 2010). A group of limb-independent cells was only found in the posterior parietal cortex of cats that discharged related to the lead limb but not related to the side of the limb. Also, gait-related power fluctuations in the posterior parietal area were not as robust as in the sensorimotor area. One potential explanation is that a smaller portion of the local neural population may be rhythmically modulated during locomotion and only engaged when more precise control of whole-body movement is needed (Andujar et al., 2010).

### 4.4. Use of visual information during uneven terrain walking

Visual information is critical for people to plan their movement when walking over uneven terrain. This is evidenced by a greater theta, and lower alpha and beta band spectral power at the occipital area during uneven terrain walking compared to flat terrain (Fig. 8). In addition, we also observed gait-related theta synchronization and gamma synchronization in all levels of uneven terrain versus flat terrain (Supplementary Fig. 4). A visual stimulus, particularly a moving stimulus, leads to changes in gamma band activity in the occipital area (Fan et al., 2007; Muthukumaraswamy & Singh, 2013). We did not instruct participants where they should look while walking, but the rigid, colored pucks were at least in their peripheral vision and may have induced changes in gamma band activity. EEG signals in the gamma band during walking are often contaminated by muscle artifacts such as neck muscle activity. Still, the gamma activity cannot be fully attributed to muscle artifacts because gait-related spectral power fluctuations in the gamma band within the occipital area differ substantially from neck muscle activity spectral power fluctuations across the gait cycle (Nordin et al., 2020).

### 4.5. Speed modulation of electrocortical dynamics

Contrary to our hypothesis, we only observed a small but significant effect of walking speed on average theta band power but not on alpha and beta band power at the sensorimotor clusters. For intra-stride spectral power fluctuations, there was significantly greater alpha and beta desynchronization during the contralateral swing phase only at 0.25 m/s versus 1.0 m/s. These findings suggest that maintaining a very slow speed (0.25 m/s) on the treadmill may require substantially more cortical processing and attentional resources for movement planning and execution in younger adults. This finding may be inconsistent with previous studies that found that fast gait speeds reduced sensorimotor alpha and beta power substantially (Nordin et al., 2020). Several reasons may explain the apparent discrepancy. First, our range of speeds was from 0.25 m/s to 1.0 m/s while previous studies focused on speeds that were higher than 0.5 m/s. For example, Nordin et al. studied the range of 0.5 m/s to 2.0 m/s (Nordin et al., 2020). The speeds we used in this study, particularly 0.25 m/s, were much slower than normal self-selected speeds in young adults (~1.3 m/s) and thus may require added cortical processing to maintain the very slow speed. Second, our study had a much larger sample size (n = 32) than previous mobile EEG studies (n = ~10). We used a similar processing pipeline, but included individual-specific head models that improved source localization (Liu et al., 2023). This enabled us to have a better estimation of sensorimotor source locations than the previous study (Nordin et al., 2020). We speculate that there might be a nonlinearity in the speed modulation of electrocortical dynamics such that only extremely slow or fast walking speeds may substantially affect electrocortical activities. This remains to be tested.

We only observed substantial intra-stride gait-related spectral power modulations with speed at the sensorimotor area but no other brain areas. Although average beta band spectral power was negatively associated with faster gait speed at the right posterior parietal cluster and mid/posterior cingulate cluster, the effect size was small. We did not find any changes in intra-stride gait-related spectral power fluctuations in the alpha or beta band at the posterior parietal, mid/posterior cingulate, premotor, and pre-supplementary motor areas as we observed during uneven terrain walking. It does not necessarily mean that other brain areas were not involved in gait speed modulation. For example, prefrontal area activation recorded with functional near-infrared spectroscopy increased in older adults during fast walking (Belli et al., 2021). However, prefrontal activity can be difficult to obtain with mobile EEG due to ocular artifacts and facial muscle activities. Better signal processing or new hardware design may be needed to identify prefrontal activity with mobile EEG. Together, our results suggest that there might be distinct neural processes that assist balance control during uneven terrain walking and adjusting gait speeds.

### 4.6. Limitations

There are several limitations to our study. The range of walking speeds selected for the speed condition was slower than the typical self-selected treadmill walking speed in young adults (Liu et al., 2022; Nagano et al., 2013). We did not collect walking trials with speeds over 1.0 m/s or with self-selected treadmill speeds for young adults because this study was part of a larger study that also aimed to recruit many older adults (>70 yrs old) who were less likely to be able to walk more than 1.0 m/s (Nagano et al., 2013). To allow for comparison between young and older adults, the walking speeds were set from 0.25 m/s to 1.0 m/s. It is also important to note that we focused on cortical areas. Electrical activity in subcortical areas and at the cerebellum is rarely to be identified with scalp EEG during human walking. We identified a cluster in the caudate area with the help of the individual-specific head model, but our focus was on cortical spectral power fluctuations. Thus, the current paper only reported results in the caudate cluster rather than have a hypothesis about how activities at caudate would change with terrain unevenness and speed. Additionally, in our study, we used individual-specific head models and high-density EEG to enhance the accuracy of EEG source localization. However, it is important to acknowledge that, even with these advanced techniques, EEG source localization can exhibit errors up to 10 mm to 20 mm for superficial cortical sources, as evidenced by previous studies validating EEG source locations (Akalin Acar & Makeig, 2013; Baillet et al., 2001; Barborica et al., 2021; Christmann et al., 2002; Cohen & Cuffin, 1991; Klamer et al., 2015; Lascano et al., 2014; Leahy et al., 1998; Megevand et al., 2014; Seeber et al., 2019; Song et al., 2015). Therefore, when interpreting dipole locations in this current study, it is essential to proceed with caution due to the inherent spatial resolution limitations.

Also, future studies may investigate gaze control during uneven terrain walking with brain dynamics. Visual control strategies can change based on terrain. A recent study found that natural uneven terrain led participants to look two steps forward while maintaining a constant ahead-looking window to gather information about their surroundings and adjust their gait pattern to maintain balance (Matthis et al., 2018). Treadmill locomotion places a limit on the forward distance available for gazing which may affect motor planning for foot placement.

Another consideration is that differences in power spectral densities between uneven terrains versus flat terrain may be affected by single and double support phase duration across conditions. However, differences in single and double support phases cannot account for all observed differences in power spectral density across conditions. For example, on average, we found a ~5% decrease in double support time comparing the high terrain and flat terrain. As a comparison, we found a ~45% decrease in beta power in the sensorimotor cluster and ~50% decrease in alpha and beta power in the posterior parietal clusters comparing high terrain versus flat (Figs. 5–6). Therefore, it is likely that variations in single and double support phase duration are not the main contributor to changes in power spectral densities across conditions. Instead, changes in power spectral density are related to greater cortical involvement during uneven terrain walking versus flat.

## Supplementary Material

Supplement

## Figures and Tables

**Table 1.** Statistical results from the pairwise comparison examining the effects of terrain unevenness on each dependent variable.

Dependent variable	Condition 1	Condition 2	DF	t Value	p Value	pFDR-_adjusted_ Value

Step Duration (seconds)	flat	low	123	1.08	0.28	0.34
	flat	med	123	−0.39	0.7	0.7
	flat	high	123	−3.05	0.003	**0.008**
	low	med	123	−1.5	0.14	0.22
	low	high	123	−4.1	<0.001	**<0.001**
	med	high	123	−2.7	0.0087	**0.017**
Step Duration CoV (%)	flat	low	123	11.7	<0.001	**<0.001**
	flat	med	123	17.8	<0.001	**<0.001**
	flat	high	123	24.3	<0.001	**<0.001**
	low	med	123	6.09	<0.001	**<0.001**
	low	high	123	12.6	<0.001	**<0.001**
	med	high	123	6.5	<0.001	**<0.001**
Anteroposterior excursion CoV (%)	flat	low	123	4.1	<0.001	**<0.001**
	flat	med	123	7.6	<0.001	**<0.001**
	flat	high	123	9.9	<0.001	**<0.001**
	low	med	123	3.5	<0.001	**<0.001**
	low	high	123	5.8	<0.001	**<0.001**
	med	high	123	2.3	0.026	**0.026**
Mediolateral excursion CoV (%)	flat	low	123	2.6	0.01	**0.015**
	flat	med	123	4.1	<0.001	**<0.001**
	flat	high	123	6.3	<0.001	**<0.001**
	low	med	123	1.5	0.15	0.15
	low	high	123	3.7	<0.001	**<0.001**
	med	high	123	2.2	0.026	**0.031**

Bold values indicated significance after adjusting for multiple comparisons.

**Table 2.** Montreal Neurological Institute (MNI) coordinates, and anatomical atlas labels for regions of interest (ROIs).

Cluster centroid	Color	No. of ICs	MNI coordinates	Anatomical atlas labels

Sensorimotor (R)	Red	21	[33 −22 57]	Primary Motor R^d^
Sensorimotor (L)	Orange	28	[−28 −33 60]	Primary Somatosensory L^d^
Premotor (R)	Medium blue	24	[20 10 37]	No label^b,d^
Pre-supplementary motor (L)	Purple	24	[−6 11 55]	Pre-supplementary Motor L^d^
Posterior parietal (R)	Green	28	[34 −60 39]	Angular R^b^
Posterior parietal (L)	Yellow	27	[−28 −67 29]	Angular L^b^
Occipital	Cyan (light blue)	20	[22 −83 23]	Occipital Sup R^b^
Mid/Posterior Cingulate	Navy	24	[1 −31 36]	Mid Cingulate R^b^ Posterior Cingulate R^c^
Caudate	Lime	20	[−20 −1 18]	No label^b^
Temporal (L)	Light Gray	17	[48 −44 −9]	Temporal Inf L^b^
Temporal (R)^a^	Not shown	12	[40 −49 −1]	No label^b^
Precuneus^a^	Not shown	15	[3 −73 52]	Precuneus R^b^

a The clusters are not shown in Figure 4 because they do not have enough ICs.

b Anatomical location of the cluster centroid was labeled based on Tzourio-Mazoyer et al. (2002).

c Anatomical location of the cluster centroid was labeled based on Papademetris et al. (2006).

d Anatomical location of the cluster centroid was labeled based on Mayka et al. (2006).

## Data Availability

Data are available via OpenNeuro: https://openneuro.org/datasets/ds004625/versions/1.0.2
