# Supplementary material for: Electrical brain activity during human walking with parametric variations in terrain unevenness and walking speed
Source: Imaging Neurosci (Camb). 2024 Feb 22;2:imag-2-00097. doi: 10.1162/imag_a_00097 (PMC11845229; doi:10.1162/imag_a_00097)
Supplement: Supplementary Material [file imag_a_00097-supp.pdf]

Supplementary Figures

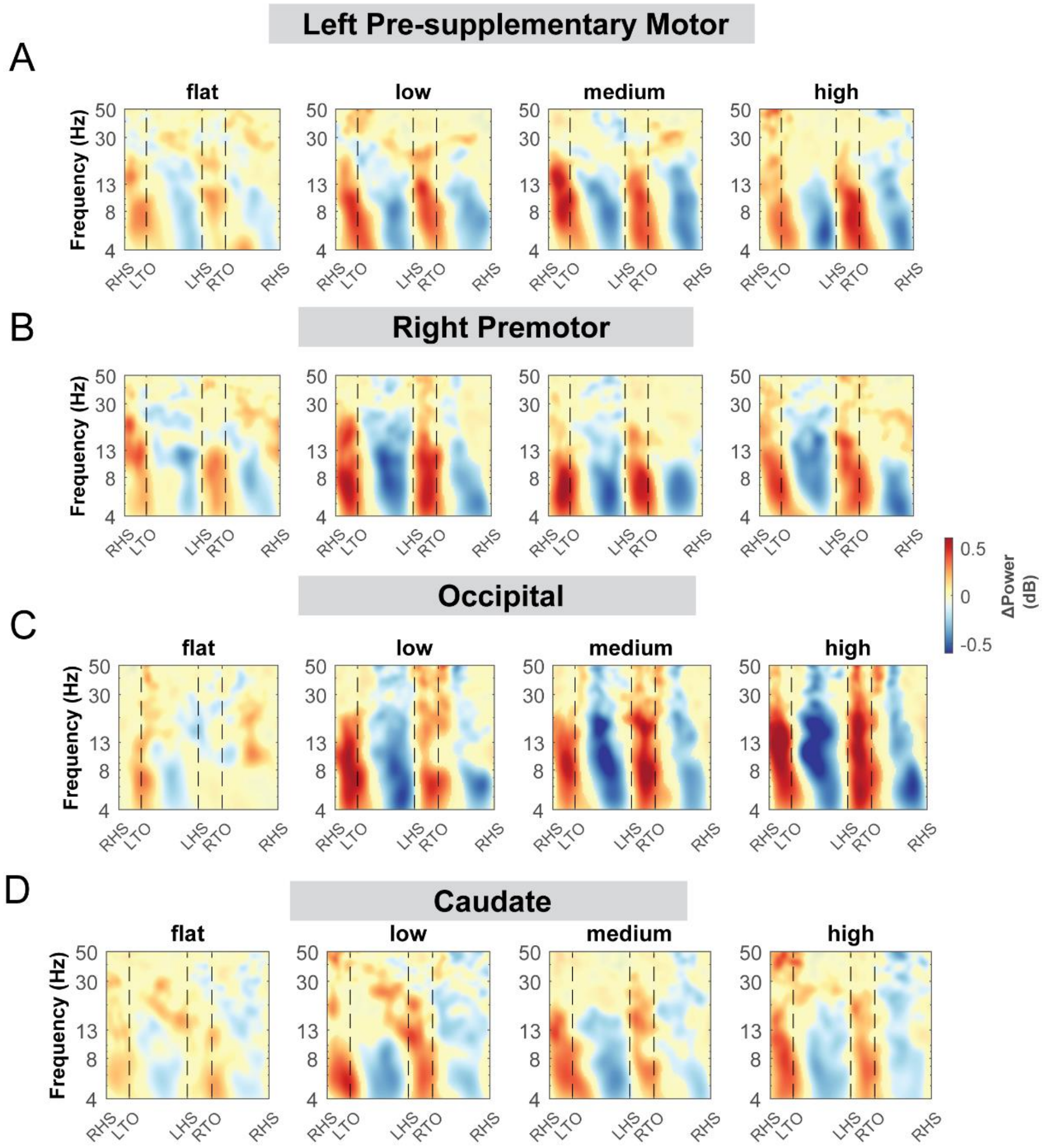

Supplementary Figure 1. ERSPs at the left pre-supplementary motor (A), right premotor, occipital (C), and caudate clusters (D) with respect to the average of each condition at different terrains. The x-axes of the ERSPs are time in gait cycle (RHS: right heel strike; LTO: left toe off; LHS: left heel strike; RTO: right toe off). All unmasked colors are statistically significant spectral power fluctuations relative to the mean power within the same condition. Colors indicate significant increases (red, synchronization) and decreases (blue, desynchronization) in spectral power from the average spectrum for all gait cycles to visualize intra-stride changes in the spectrograms. These data are significance masked ($p < 0.05$) through nonparametric bootstrapping with multiple comparison correction using false discovery rate.

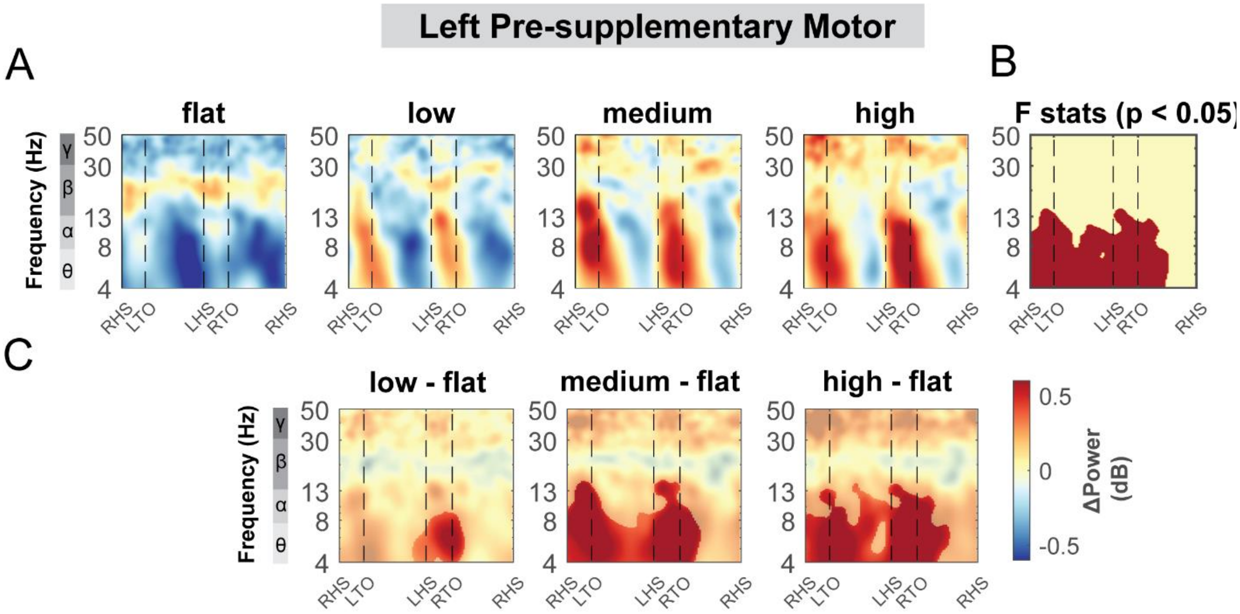

Supplementary Figure 2: ERSPs at the left pre-supplementary motor clusters with respect to the grand average of all conditions and with respect to flat terrain condition. (**A**) Averaged ERSP at different terrain at the left pre-supplementary motor cluster. Red indicated spectral power increases (neural synchronization) and blue indicated spectral power decreases (neural desynchronization) relative to the grand average of all conditions. Vertical dashed lines indicated gait events. RHS: right heel strike; LTO: left toe off; LHS: left heel strike; RTO: right toe off. (**B**) Significant effect of terrain on ERSPs across gait cycle with non-parametric statistics, with red indicating significance ($p<0.05$). (**C**) ERSPs with respect to flat condition at the left pre-supplementary motor cluster. Regions that are not significantly different from flat condition have a semi-transparent mask as determined by cluster-based permutation.

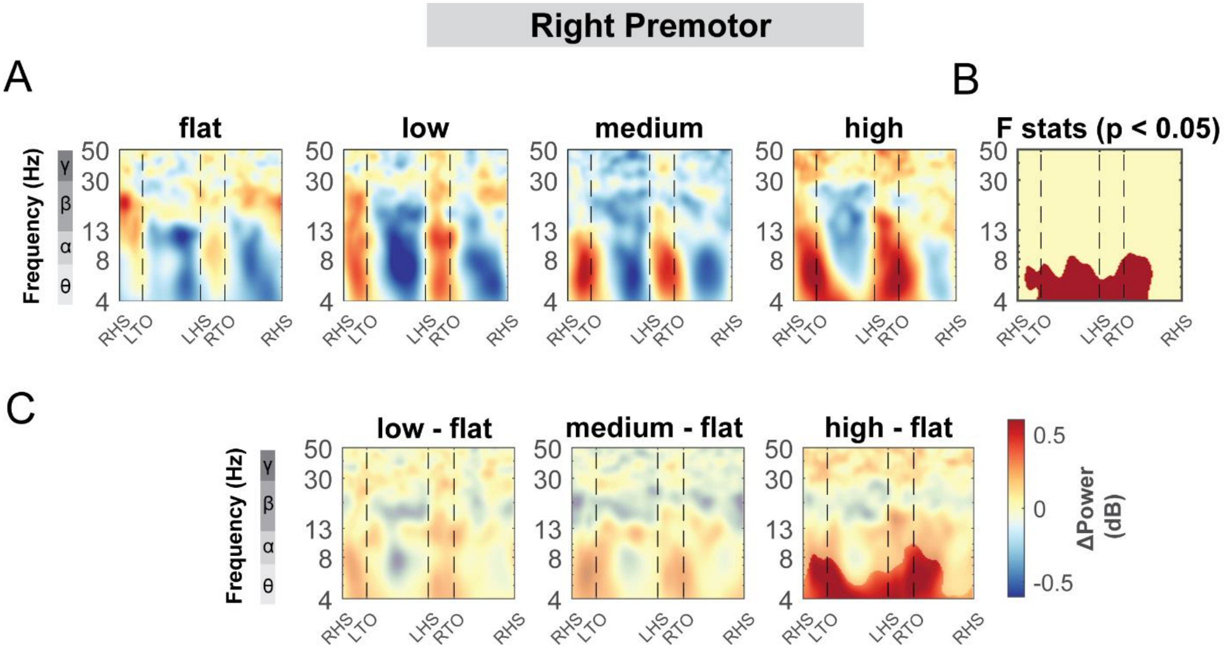

Supplementary Figure 3: ERSPs at the right premotor cluster with respect to the grand average of all conditions and with respect to flat terrain condition. (**A**) Averaged ERSP at different terrain at the right premotor cluster. Red indicated spectral power increases (neural synchronization) and blue indicated spectral power decreases (neural desynchronization) relative to the grand average of all conditions. Vertical dashed lines indicated gait events. RHS: right heel strike; LTO: left toe off; LHS: left heel strike; RTO: right toe off. (**B**) Significant effect of terrain on ERSPs across gait cycle with non-parametric statistics, with red indicating significance ($p<0.05$). (**C**) ERSPs with respect to flat condition at the right premotor cluster. Regions that are not significantly different from flat condition have a semi-transparent mask as determined by cluster-based permutation.

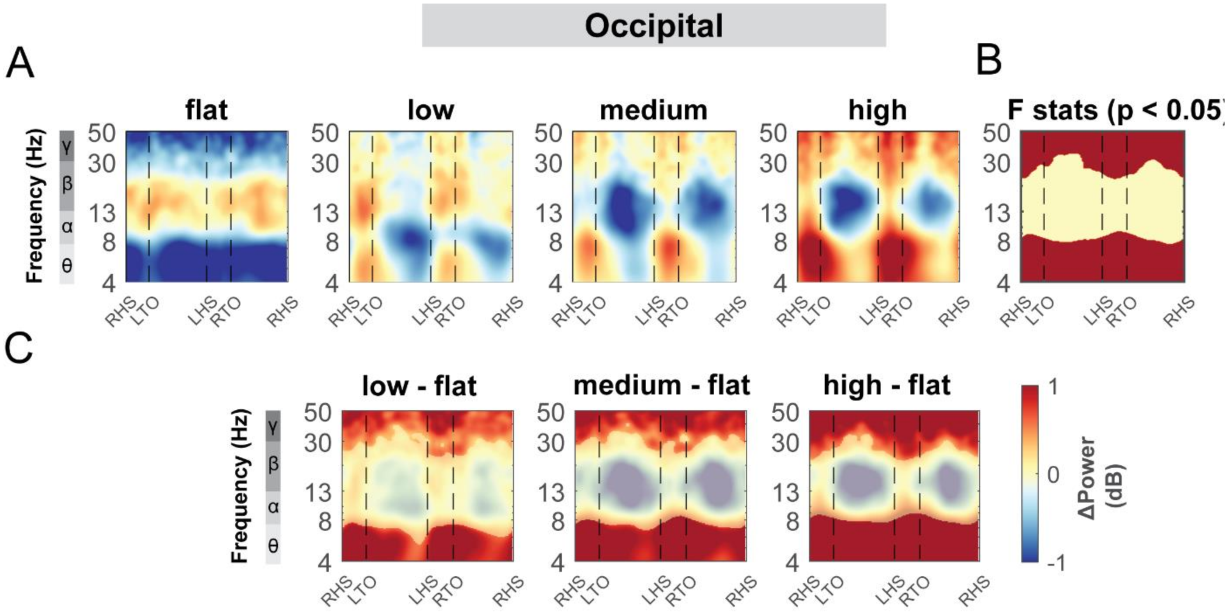

Supplementary Figure 4: **ERSPs at the occipital cluster with respect to the grand average of all conditions and with respect to flat terrain condition.** (**A**) Averaged ERSP at different terrain at the occipital cluster. Red indicated spectral power increases (neural synchronization) and blue indicated spectral power decreases (neural desynchronization) relative to the grand average of all conditions. Vertical dashed lines indicated gait events. RHS: right heel strike; LTO: left toe off; LHS: left heel strike; RTO: right toe off. (**B**) Significant effect of terrain on ERSPs across gait cycle with non-parametric statistics, with red indicating significance ($p < 0.05$). (**C**) ERSPs with respect to flat condition at the occipital cluster. Regions that are not significantly different from flat condition have a semi-transparent mask as determined by cluster-based permutation.

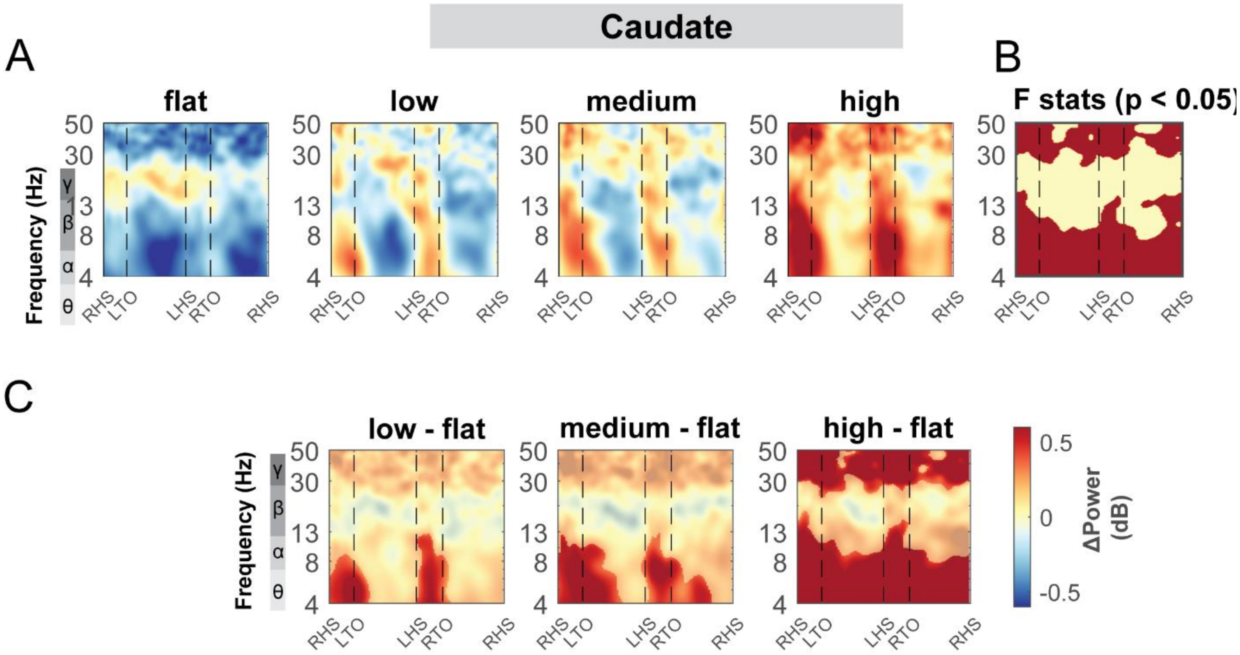

Supplementary Figure 5: ERSPs at caudate cluster with respect to the grand average of all conditions and with respect to flat terrain condition. (**A**) Averaged ERSP at different terrain at the caudate cluster. Red indicated spectral power increases (neural synchronization) and blue indicated spectral power decreases (neural desynchronization) relative to the grand average of all conditions. Vertical dashed lines indicated gait events. RHS: right heel strike; LTO: left toe off; LHS: left heel strike; RTO: right toe off. (**B**) Significant effect of terrain on ERSPs across gait cycle with non-parametric statistics, with red indicating significance (p<0.05). (**C**) ERSPs with respect to flat condition at the caudate cluster. Regions that are not significantly different from flat condition have a semi-transparent mask as determined by cluster-based permutation.

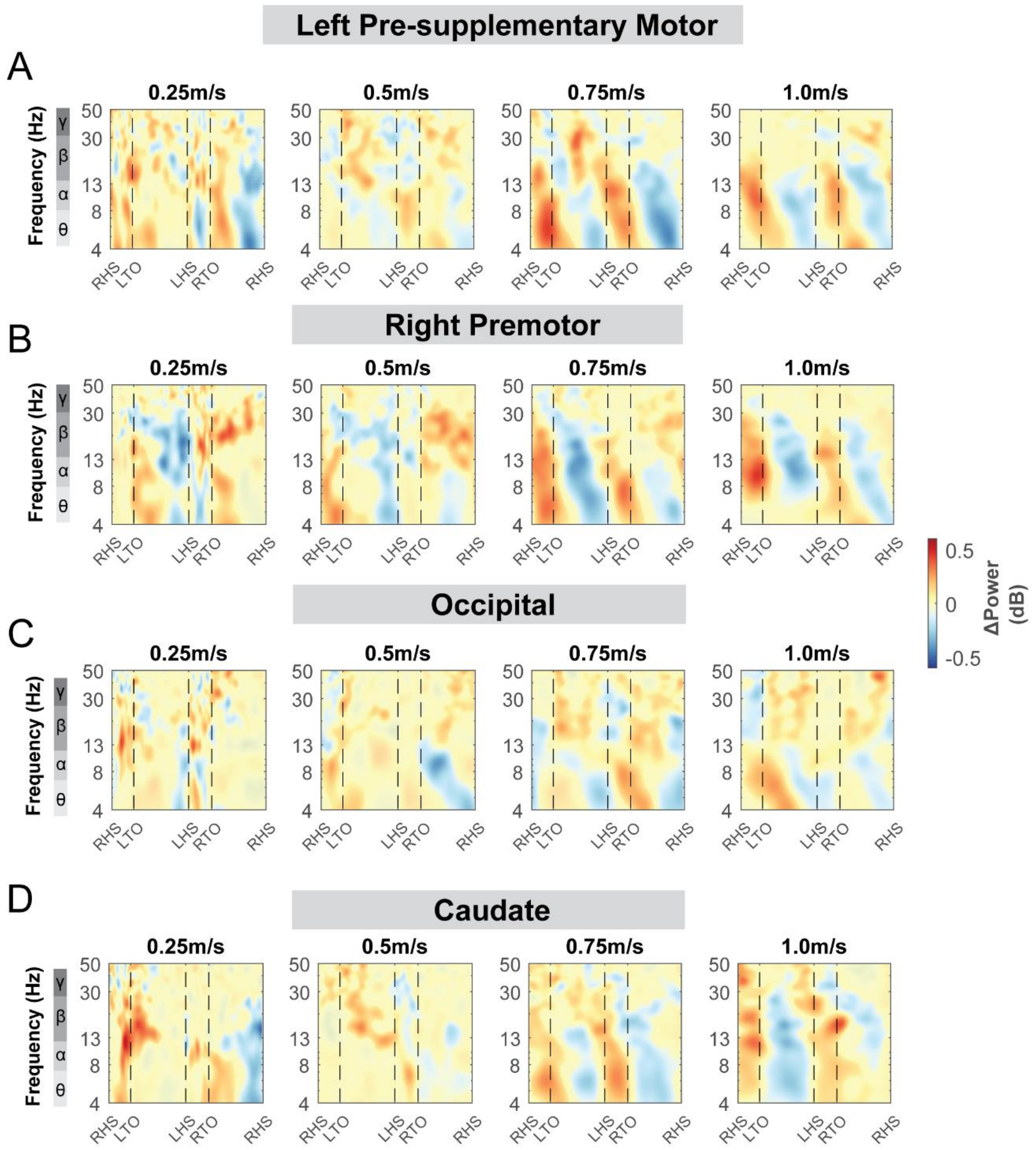

Supplementary Figure 6. ERSPs at the left pre-supplementary motor (A), right premotor (B), occipital (C), and caudate areas (D) with respect to the average of each condition at different speeds. The x-axes of the ERSPs are time in gait cycle (RHS: right heel strike; LTO: left toe off; LHS: left heel strike; RTO: right toe off). All unmasked colors are statistically significant spectral power fluctuations relative to the mean power within the same condition. Colors indicate significant increases (red, synchronization) and decreases (blue, desynchronization) in spectral power from the average spectrum for all gait cycles to visualize intra-stride changes in the spectrograms. These data are significance masked ($p<0.05$) through nonparametric bootstrapping with multiple comparison correction using false discovery rate.

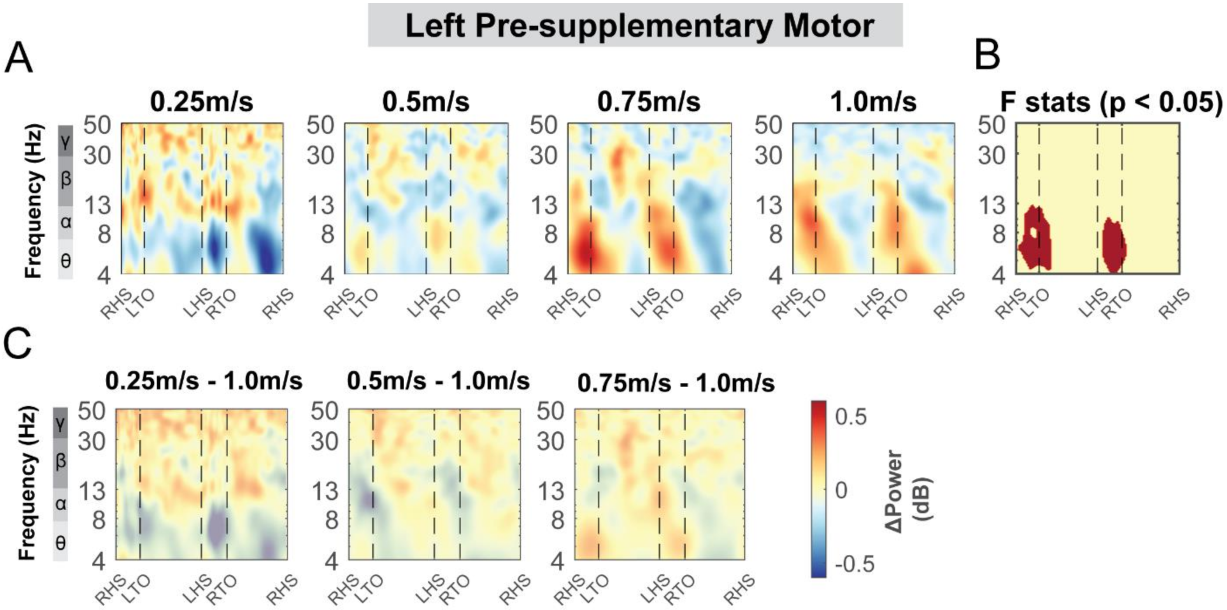

Supplementary Figure 7: **ERSPs at the left pre-supplementary motor area with respect to the grand average of all conditions and with respect to the 1.0 m/s speed condition.** (**A**) Averaged ERSP at different speeds at the left pre-supplementary cluster. Red indicated spectral power increases (neural synchronization) and blue indicated spectral power decreases (neural desynchronization) relative to the grand average of all conditions. Vertical dashed lines indicated gait events. RHS: right heel strike; LTO: left toe off; LHS: left heel strike; RTO: right toe off. (**B**) Significant effect of terrain on ERSPs across gait cycle with non-parametric statistics, with red indicating significance ($p<0.05$). (**C**) ERSPs with respect to 1.0 m/s speed condition. Regions that are not significantly different from 1.0 m/s condition have a semi-transparent mask.

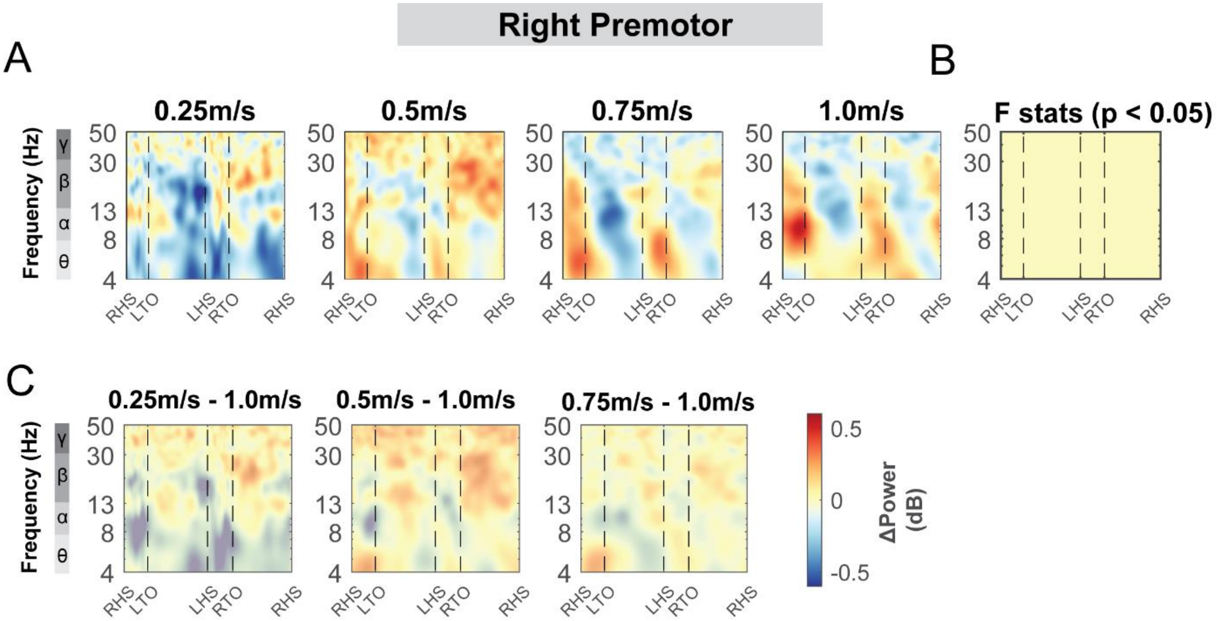

Supplementary Figure 8: ERSPs at the right premotor cluster with respect to the grand average of all conditions and with respect to the 1.0m/s speed condition. (**A**) Averaged ERSP at different speeds at the right premotor cluster. Red indicated spectral power increases (neural synchronization) and blue indicated spectral power decreases (neural desynchronization) relative to the grand average of all conditions. Vertical dashed lines indicated gait events. RHS: right heel strike; LTO: left toe off; LHS: left heel strike; RTO: right toe off. (**B**) Significant effect of terrain on ERSPs across gait cycle with non-parametric statistics, with red indicating significance (p<0.05). (**C**) ERSPs with respect to 1.0 m/s speed condition. Regions that are not significantly different from 1.0 m/s condition have a semi-transparent mask.

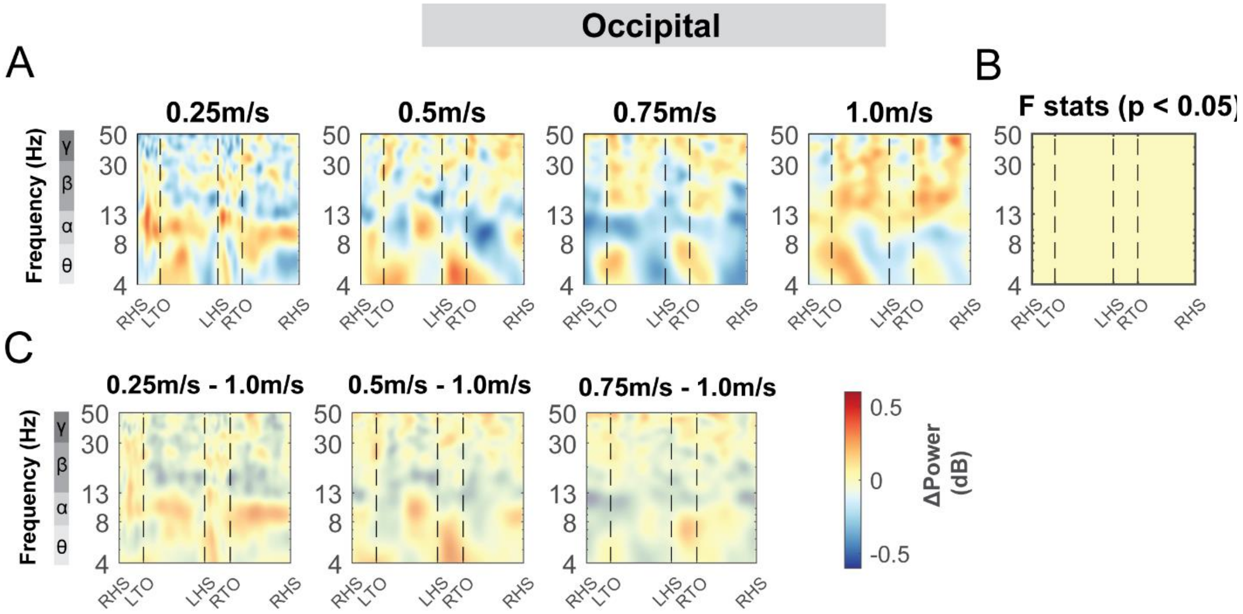

Supplementary Figure 9: **ERSPs at the occipital cluster with respect to the grand average of all conditions and with respect to the 1.0m/s speed condition**. (**A**) Averaged ERSP at different speeds at the occipital cluster. Red indicated spectral power increases (neural synchronization) and blue indicated spectral power decreases (neural desynchronization) relative to the grand average of all conditions. Vertical dashed lines indicated gait events. RHS: right heel strike; LTO: left toe off; LHS: left heel strike; RTO: right toe off. (**B**) Significant effect of terrain on ERSPs across gait cycle with non-parametric statistics, with red indicating significance ($p<0.05$). (**C**) ERSPs with respect to 1.0 m/s speed condition. Regions that are not significantly different from 1.0 m/s condition have a semi-transparent mask.

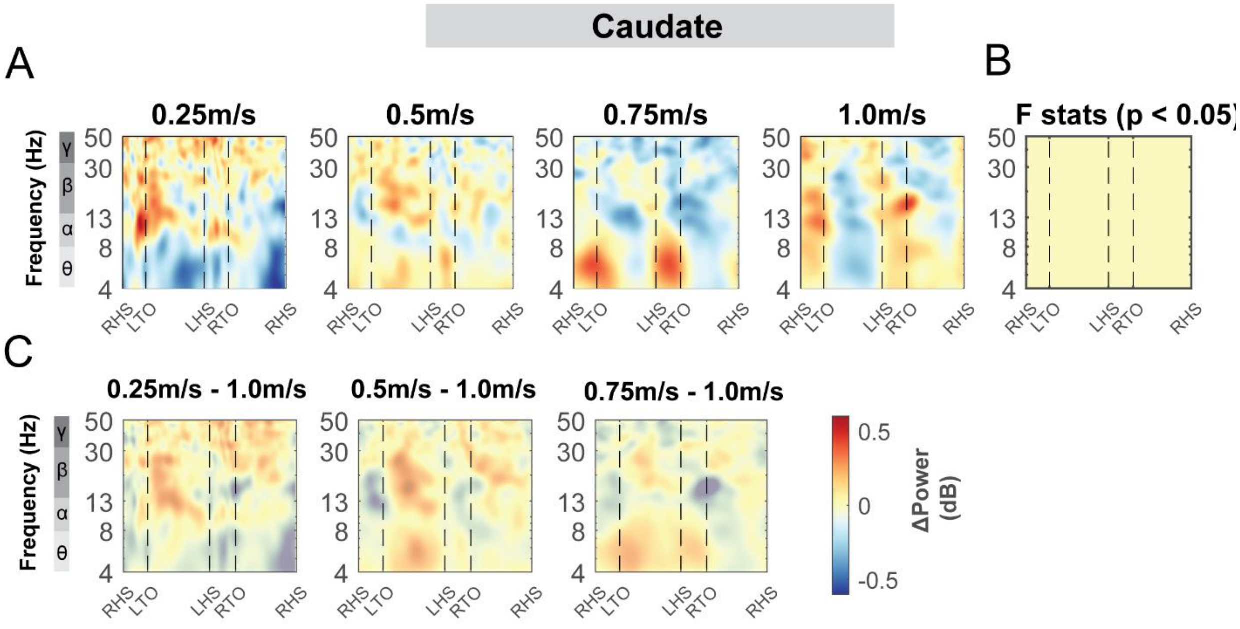

Supplementary Figure 10: ERSPs at the caudate cluster with respect to the grand average of all conditions and with respect to the 1.0m/s speed condition. (**A**) Averaged ERSP at different speeds at caudate cluster. Red indicated spectral power increases (neural synchronization) and blue indicated spectral power decreases (neural desynchronization) relative to the grand average of all conditions. Vertical dashed lines indicated gait events. RHS: right heel strike; LTO: left toe off; LHS: left heel strike; RTO: right toe off. (**B**) Significant effect of terrain on ERSPs across gait cycle with non-parametric statistics, with red indicating significance ($p<0.05$). (**C**) ERSPs with respect to 1.0 m/s speed condition. Regions that are not significantly different from 1.0 m/s condition have a semi-transparent mask.
